# Computational, In Vitro, and In Vivo Evaluation of Benzimidazole–Pyrazole Hybrid Scaffolds as Multi‐Target Anti‐Ulcer Agents

**DOI:** 10.1002/cbdv.71698

**Published:** 2026-09-15

**Authors:** Hafiz Aamir Ali Kharl, Abdul Malik, Humaira Nadeem, Mohammad Shamsul Ola, Sara Aiman, Arif Ullah Khan, Muhammad Akmal Farooq, Humaira Muzaffar, Syed Muzzammil Masaud

**Affiliations:** ^1^ Riphah Institute of Pharmaceutical Sciences Riphah International University Islamabad Pakistan; ^2^ Department of Pharmaceutics, College of Pharmacy King Saud University Riyadh Saudi Arabia; ^3^ Department of Biochemistry, College of Science King Saud University Riyadh Saudi Arabia; ^4^ Guangdong Provincial Key Laboratory of Medical Immunology and Molecular Diagnostics, Dongguan Affiliated Hospital Guangdong Medical University Dongguan China; ^5^ Department of Pharmacy, Faculty of Health & Pharmaceutical Sciences University of Agriculture Faisalabad Pakistan; ^6^ Department of Physiology Government College University Faisalabad Faisalabad Pakistan; ^7^ Department of Allied Health Sciences The Saifee Burhani University Karachi Pakistan

**Keywords:** anti‐inflammatory, antioxidant, benzimidazole‐pyrazole hybrids, COX‐2, H^+^/K^+^ ATPase, MD simulation, molecular docking, peptic ulcer

## Abstract

Peptic ulcer disease (PUD) continues to be an important gastrointestinal condition attributed to *Helicobacter pylori* infection, usage of nonsteroidal anti‐inflammatory drugs, oxidative stress, and inflammatory mediators. Although there are available treatments for this condition, side effects, relapse, and safety issues call for development of new multi‐target anti‐ulcer compounds. In this research, a group of benzimidazole‐pyrazole analogs (5a–5j) was designed, synthesized, and studied with the aid of computational and experimental methods. Molecular docking into H^+^/K^+^‐ATPase (PDB ID: 5YLU) and COX‐2 (PDB ID: 3LN1) enzymes predicted high affinity of these compounds with docking scores of −9.4 and −9.3 kcal/mol for compounds 5i and 5e, respectively, higher than those obtained for the reference drugs omeprazole and celecoxib. The inhibition of the gastric H^+^/K^+^‐ATPase enzyme was also observed in vitro, with compounds 5i and 5e having better inhibitory effects. Studies on the ethanol and indomethacin‐induced gastric ulceration models demonstrated good inhibitory effects of these compounds. Biochemical analysis showed that these analogs significantly decreased the levels of TNF‐α, COX‐2, myeloperoxidase, and malondialdehyde and increased prostaglandin E_2_. Acute oral toxicity studies indicated no observable toxicity at 100 mg/kg. Collectively, these findings identify compounds 5i and 5e as promising multifunctional gastroprotective candidates warranting further pharmacological investigation.

## Graphical abstract

The hybrid pharmacophore approach involves the combination of benzimidazole‐pyrazole moieties to form multi‐functional anti‐ulcer compounds. Molecular modeling and biological evaluations show significant proton pump inhibition, anti‐inflammatory action, antioxidant property, and remarkable prevention of gastric ulcers.

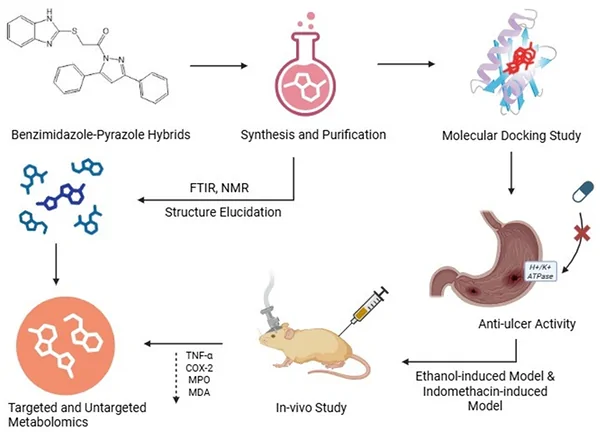

## 1 Introduction

**FIGURE 1:**
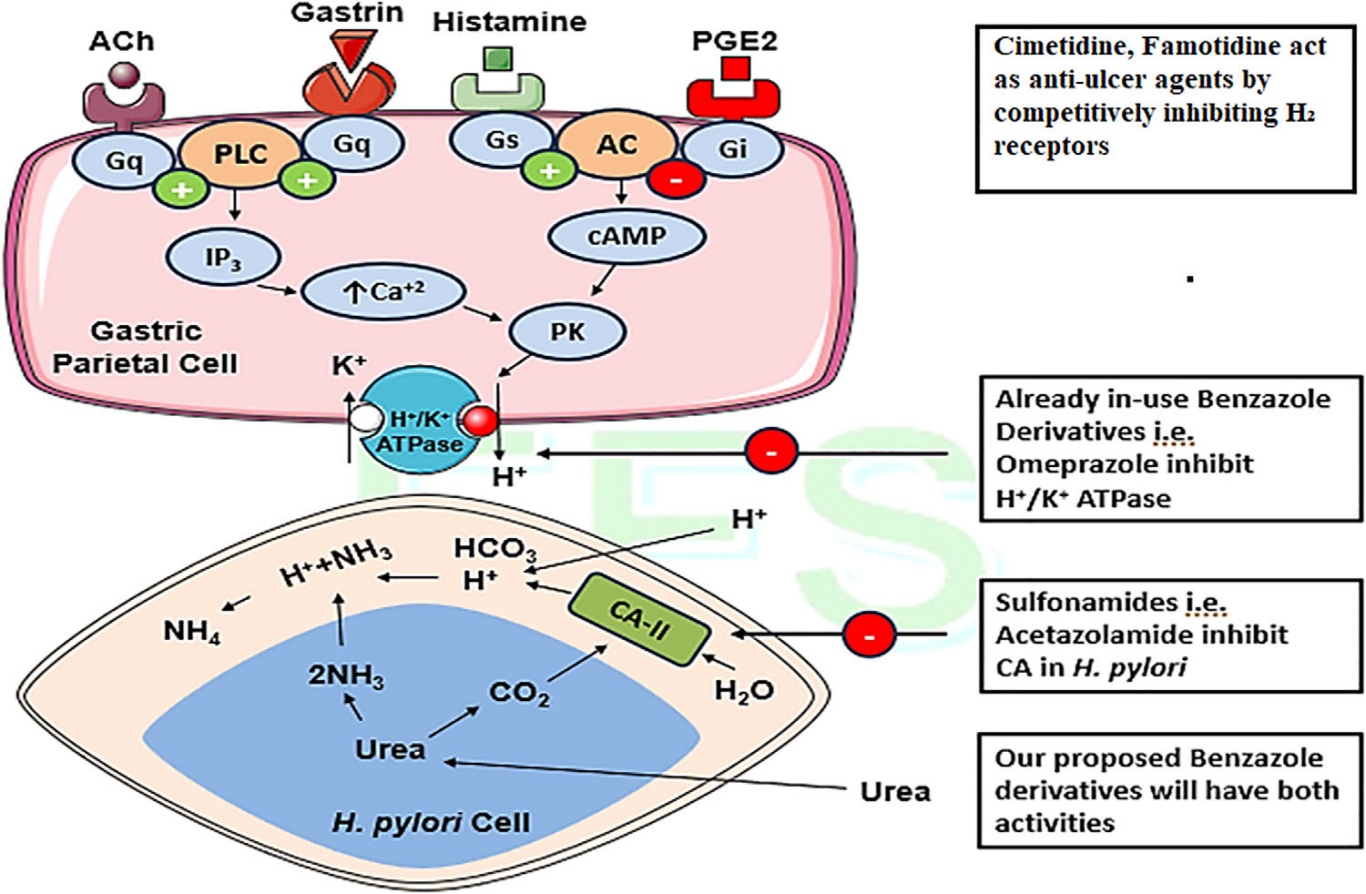
Key regulators of Gastric acid secretion and the survival mechanisms of *Helicobacter pylori*. Existing therapies target either H^+^/K^+^‐ATPase or Carbonic Anhydrase, the proposed compounds are intended to block both pathways, providing enhanced anti‐ulcer and anti‐*H. pylori* efficacy.

The global rate and proportion of peptic ulcer cases enhanced by 11.1% and 8.8%, respectively, between 1990 and 2021. On the other hand, the number of deaths and DALYs (Disability‐Adjusted Life Years) dropped by 15.94% and 27.8%, respectively, during the same period. The global age‐standardized rates (ASRs) for occurrence, mortality, prevalence, and DALYs related to peptic ulcer disease reduced by 40.3%, 41.1%, 61.5%, and 63.1%, respectively, between 1990 and 2021 [1]. Peptic ulcer is nowadays very common and mainly occurs due to an imbalance between mucosal protecting (mucus, bicarbonate, Prostaglandin E2 and I2) and damaging (acid, pepsin) factors. Acid release is a physiologically vital process in the stomach resulting in activation of pepsinogen to instigate digestive process and also protects against microorganisms including bacteria to establish a stable environment of stomach [2]. Acetyl choline, histamine and gastrin are three endogenous positive regulators of acid secretion whereas Prostaglandins (PG E2 and I2) work as negative modulators [3] (Figure 1).

**FIGURE 2:**
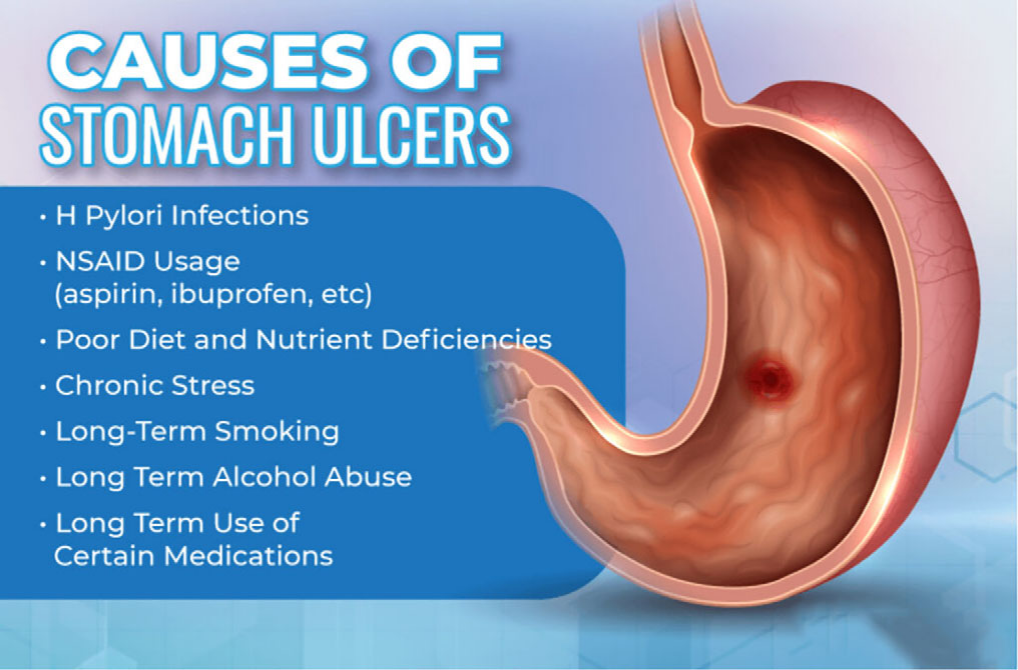
*Helicobacter pylori* infection, chronic use of NSAIDs, excess secretion of acid from the stomach, intake of alcohol, smoking, and stress‐all contribute to peptic ulcers. These factors disrupt the balance between mucosal defense and aggressive agents, leading to ulcer formation.

Inflammation is a pathological state characterized mainly by a multicause organic reaction of body tissues to physical and biochemical excitants. This process involves immune cells, molecular intermediaries, and blood vessels to clear injury and initiate tissue healing, but chronic inflammation can also herald the onset of many diseases [4, 5, 6]. Neutrophils have critical roles in the development of injury and inflammation in a range of tissues, including gastric mucosa. Inflammatory cytokines, such as TNF‐α, are critical mediators of ulcer recurrence. TNF‐α enhances expression of adhesion molecules on both leukocytes and endothelial cells and cytokines, resulting in neutrophil infiltration into scarred mucosa. Gastric acid also plays an important role in recurrence of gastric ulcer [7, 8, 9]. The Gram‐negative bacterium *Helicobacter pylori* resides between the gastric epithelium and the mucous layer, uniquely adapted to survive the harsh acidic conditions of the stomach. Its presence triggers an inflammatory response involving neutrophils, lymphocytes, plasma cells, and macrophages in the mucosal layer, ultimately leading to epithelial cell damage and dysfunction [10]. *Helicobacter pylori* infection of the gastroduodenal mucosa stimulates the local production of several proinflammatory and immunoregulatory cytokines, causing neutrophil infiltration, specific T‐ and B‐cell responses, and the appearance of gastric lymphoid follicles. Tumor necrosis factor‐alpha (TNF‐α) enhances, and *H. pylori* strains with the CagA phenotype directly induce IL‐8 production by gastric epithelial cells. Current in vivo and ex vivo studies reveal that the presence of CagA‐and VacA‐expressing *H. pylori* strains is associated with stronger inflammatory responses and more severe mucosal injury [11]. Non‐steroidal anti‐inflammatory drugs (NSAIDs) damage the gastroduodenal mucosa through both local and systemic pathways; however, the primary mechanism involves the systemic inhibition of cyclooxygenase‐1 (COX‐1), which suppresses the production of protective prostaglandins. The COX hypothesis is supported by surveys showing that coadministration of exogenous prostaglandins reduces mucosal injury [12]. NSAIDs initiate mucosal injury by disrupting mucus phospholipids or the cell membrane and by interfering with mitochondrial oxidative phosphorylation [13]. Severe physiological stress such as that experienced during critical illness requiring intensive care is a recognized cause of peptic ulcers, commonly referred to as stress ulcers [14]. Additionally, missing meals puts the lining of the stomach directly in contact with the gastric acid as a result of which irritation and formation of ulcers can take place over a period of time. During gastric ulcers, pain in the abdomen worsens after intake of food. Alcohol consumption and smoking also causes ulcer. Alcohol, through its chronic consumption, destroys gastric mucosal barriers by stripping off COX‐1 receptor enzymes which lessen the secretion of cytoprotective prostaglandin (PGE2). Cigarette smoking also interferes with circulating epidermal growth factor and increases the level of free radical secretion in gastric mucosa resulting in ulcer [15] (Figure 2).

To date, various challenges on drug discovery, synthesis, cost, and time can be overcome by the use of computer‐aided drug design, also called molecular modeling. This is a technique that uses iterative in silico simulations to help design and analyze potential drug candidates. Computational methods like these allow for the prediction of major characteristics of a potential drug, including toxicity, efficacy, biological activity, and bioavailability before experimentation in vitro or in vivo is conducted [16].

**FIGURE 3:**
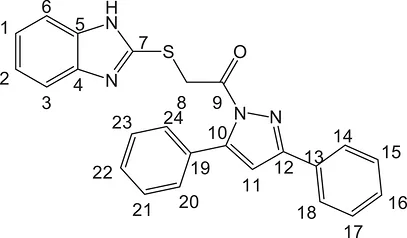
Structure of Benzimidazole‐pyrazole hybrids.

Chalcones are not only the privileged scaffolds of chemical synthesis, but also have various pharmacodynamics and clinical activities. Clinical research proved that they have significant bioavailability and maximum tolerance in the human body [17]. So, globally, research laboratories are focusing on the synthesis of several chalcone analogues for the development of new more potent drugs. Nitrogen‐containing heterocyclic compounds are an important class of chemical entities found in numerous biologically active molecules, natural products, and pharmaceutical agents [18, 19]. Structures such as benzimidazole, quinoline, indole, pyrrole, and pyrazole are particularly important in medicinal and organic chemistry. Owing to the broad range of applications, the synthesis of such heterocyclic frameworks has lately become one of the central areas of interest in organic synthesis research [20]. Among the nitrogen‐containing heterocyclic compounds, isatin and hydrazide derivatives having an oxindole moiety act as a scaffold of high value in medicinal chemistry [21]. These two heterocycles synergistically often offer compounds with enhanced pharmacological profiles. Hence, such moieties may prove to be promising candidates for drug development. Benzimidazole‐pyrazole hybrids were proved to be significant enzyme inhibitors but need some research on enzyme‐specific mechanisms, SAR optimization, and in vivo evaluations [22]. Taking into account the well‐proven antiserotonergic activity of benzimidazoles and antinflammatory activity of structures containing a pyrazole core, we assumed that new benzimidazole‐pyrazole hybrids may possess a multi‐target gastroprotective activity, which includes inhibition of gastric H^+^/K^+^‐ATPase in order to inhibit acid secretion and minimize the impact of aggressive substances on the mucosa. In turn, modulation of proinflammatory cytokines such as COX‐2 and TNF‐α may affect the inflammatory pathway involved in ulcer development. In addition, reduction of the levels of biomarkers of oxidative stress, namely myeloperoxidase (MPO) and malondialdehyde (MDA), may contribute to prevention of the migration of neutrophils and the processes of lipid peroxidation. Thus, in order to verify our assumption regarding a multi‐target gastroprotective mechanism of these molecules, we used an integrated experimental approach that included molecular docking and dynamics modeling, inhibition of H^+^/K^+^‐ATPase tests, antiulcer investigations, and assessment of inflammation and oxidative stress biomarkers. By molecular modeling, including Molecular docking and MD Simulation methods, it is possible to get valuable insights about enzyme regulation and therapeutic agent development. Therefore, efforts are made to develop novel heterocyclic systems containing benzimidazole and pyrazole moieties (Figures 3 and S3).

**FIGURE 4:**
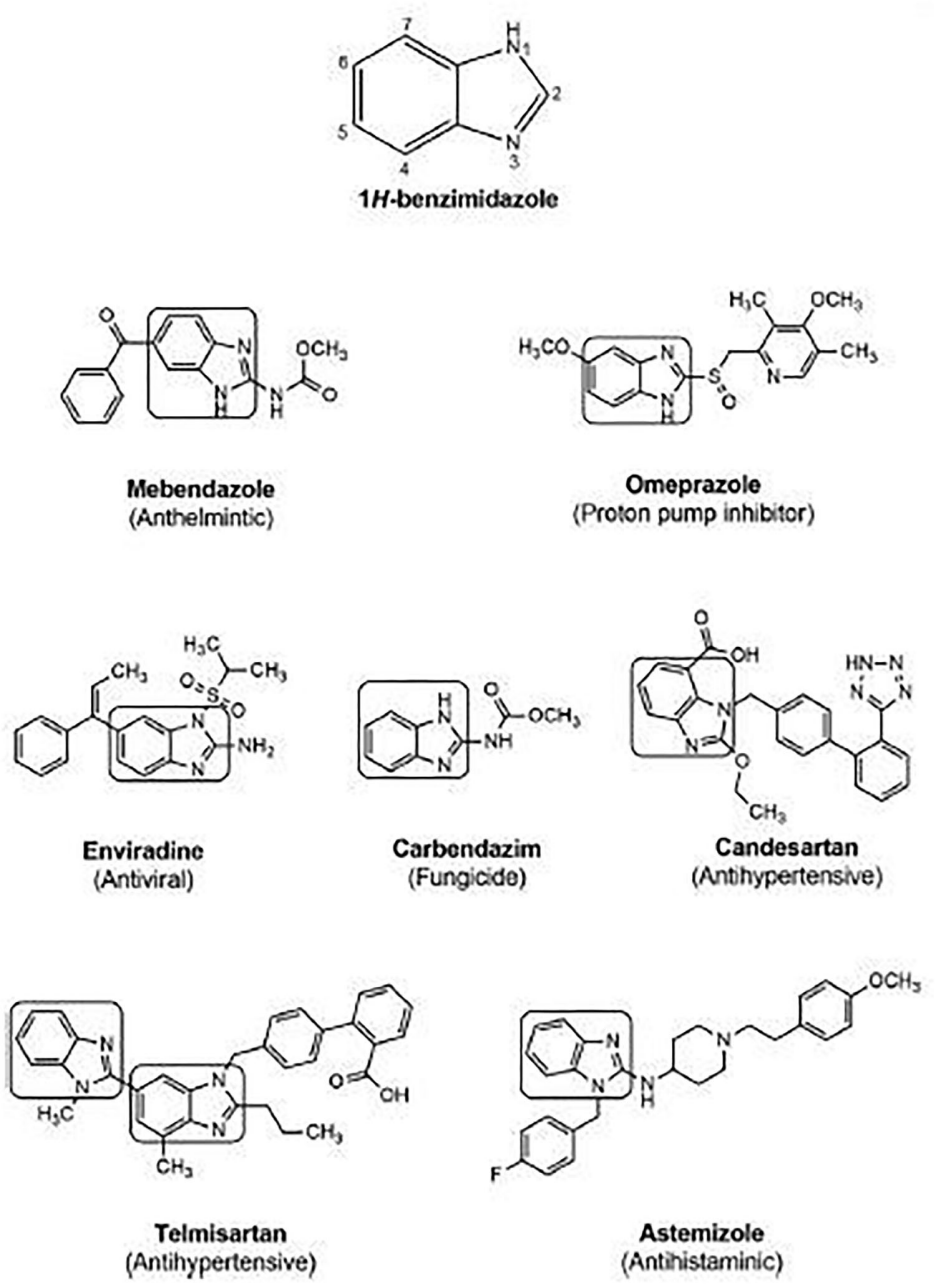
Marketed drugs containing Benzimidazole nucleus.

The benzimidazole moiety represents a part of a wide variety of pharmacological agents, such as antibiotics, antiviral, antiparasitic, antitumor agents, anti‐inflammatory drugs, antioxidants, PPIs, antihypertensives, anticoagulants, immunomodulatory agents, hormone regulators, central nervous system stimulants and depressants, antihyperlipidemic agents, and antidiabetics. This versatile structure has made it an essential scaffold for the development of novel drugs. Various substituents around the benzimidazole ring are responsible for showing its wide spectrum of biological activities. The significance of this nucleus in certain key activities, such as Angiotensin I (AT1) receptor antagonism and proton pump inhibition, has been extensively reviewed in the literature. Thus, modification of substituent groups has enabled the synthesis of a wide range of therapeutically active compounds. Examples include the anthelmintic albendazole, mebendazole, and thiabendazole; the antiviral drug enviradine; the antifungal compound carbendazim; the proton pump inhibitors omeprazole, lansoprazole, and pantoprazole; the antihypertensive agents candesartan cilexetil and telmisartan; and the anti‐allergic medication astemizole [23] (Figure 4).

## 2 Materials and Methods

### 2.1 Materials

All the drugs, chemicals and reagents utilized in this research were of high quality and purity ranging to 99% or more and were used as supplied requiring no further purification. To determine the inhibitory properties on H^+^/K^+^ ATPase the H^+^/K^+^ ATPase inhibition assay kit (Catalog No. E‐BC‐K122‐S) was purchased from Elabscience (USA). Cayman chemicals also supplied other biochemical kits. FTIR, performed on an ALPHA Eco ATR spectrophotometer was used to characterize the synthesized compound whereas melting point apparatus, Stuart melting point apparatus (Model SMP10) was used to measure the melting points. Solvent (CH_3_CN), the chemicals (OPD, CS_2_, chloroacetic acid, KOH, Et_3_N, SOCl_2_) were obtained commercially either through Sigma‐Aldrich and were purified. Solvents used in the synthetic reactions were washed according to a standard procedure. Substrates of major interest such as substituted aldehydes and ketones were purchased in Sigma‐Aldrich. The structural identification was carried by recording ^1^H‐NMR and ^13^C‐NMR spectra on Bruker DRX‐400 MHz spectrometer at 400 and 100 MHz theoretically. Chemical shift calibration was done by using TMS as an internal standard.

### 2.2 Experimental Animals

The in vivo tests was conducted male adult albino rats (Wistar strain), (weight 180–220 g). The Animal House Facility of Riphah Institute of Pharmaceutical Sciences (RIPS), Riphah International University supplied the experimental animals. They were kept in a confined laboratory environment with 4–5 animals in each cage. The temperature of the environment was standardized at 22°C ± 2°C and relative humidity of 50%–60%, 12 h of light/dark cycle. All animals were freely provided with clean water to drink, and standard food pellets were available to them. Animals were randomly assigned to treatment groups using a simple random allocation procedure. Biochemical measurements and ulcer scoring were performed by investigators blinded to treatment allocation. Sample sizes (*n* = 6/group) were selected based on previous anti‐ulcer studies employing similar experimental designs. Only one sex was used to minimize hormonal variability and reduce experimental complexity. Future studies will investigate sex‐dependent responses. The arrangement and processes performed were in accordance with the existing ethical principles of the institution, which were agreed by the RIPS Ethical Committee, Pakistan, with the reference number REC/RIPS/2021/013 approved on 12/12/2021. All experiments involving animals were carried out and published in compliance with the guidelines for Animal Research: Reporting of In Vivo Experiments (ARRIVE 2.0) and conformed to institutional ethical protocols.

### 2.3 Synthesis of Benzimidazole‐Pyrazole Hybrids (**5a–5j**)

**FIGURE 5:**
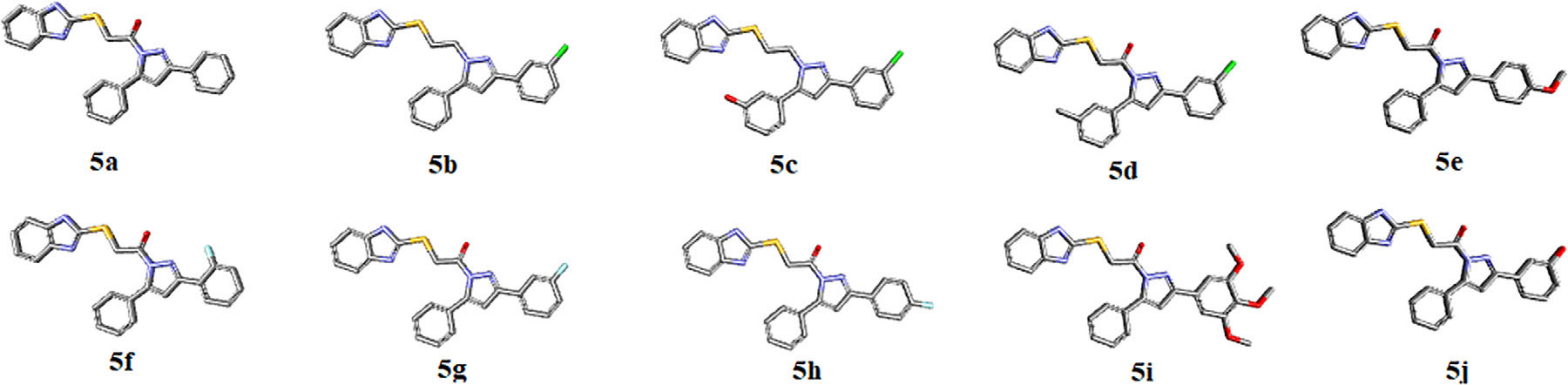
Chemical structures of the synthesized benzimidazole‐pyrazole hybrids (**5a–5j**).

Benzimidazole‐Pyrazole hybrids (**5a–5j**) were synthesized and characterized as reported [24] (Figures 5, S1, and S2) (Schemes S1 and S2).

### 2.4 Characterization of Benzimidazole‐Pyrazole Hybrids (**5a–5j**)

Characterization data of synthesized compounds is presented in Table S1 and Figures S4–S36.

### 2.5 Molecular Docking Studies

In silico studies were conducted using only free license software packages. Machine used for molecular docking was a desktop computer built of Intel core i5‐7500‐CPU @ 3.40 GHz, 3.41 GHz (gigahertz) with 16 GB (gigabyte) DDR3 RAM (Double Data Rate 3 Random Access Memory), running Windows 10 operating system. ChemSketch v2.5, DSV v17.2.0.16349, PyMOL (TM) 1.7.4.5 Edu, Schrodinger Maestro 12.5, Gromacs, AutoDockTools v1.5.6 were used for ligands and proteins preparation [25]. AutoDockVina 1.1 was used for molecular docking. All the proposed ligands were docked with the protein using AutoDock Vina. Preparation steps of ligand, protein and the procedure of docking performed are given below.

### 2.6 Grid Parameters

Grid parameters were set to focus the docking simulation on the active binding pockets of each target protein using Schrödinger Maestro v12.5. The grid box configurations for the proteins were as follows:

- **H^+^/K^+^‐ATPase (PDB ID: 5YLU)**
  - Center: X = 63.557, Y = −27.260, Z = 60.056
  - Dimensions: X = 78, Y = 50, Z = 64
- **Cyclooxygenase‐2 (COX‐2, PDB ID: 3LN1)**
  - Center: X = 18.639, Y = −52.364, Z = 54.125
  - Dimensions: X = 30, Y = 80, Z = 80

### 2.7 Preparation of Target Protein

Theoretical studies were performed to correlate the relative affinity of the ligands against the enzyme and also to understand the molecular basis of interaction of benzimidazole‐pyrazole hybrids (5a–5j) against selective proteins like H+/K+ ATPase (5YLU), Cyclooxygenase II (3LN1). The protein structure (PDB ID 5YLU & 3LN1) was downloaded from RCSB Protein Data Bank Site. DSV was used to eliminate the bound ligands and solvent molecules. This pure protein molecule was stored in the pdb format. The protein was then protonated and saved in the dockable file format, pdbqt with the help of AutoDock Tools.

### 2.8 Ligand Preparation

Ligand structures were initially sketched using ChemSketch software. The three‐dimensional geometries of these ligands were optimized and saved in MOL format. Subsequently, OpenBabel was employed to convert the ligand files from MOL to PDB format [26]. The resulting PDB files were then processed in AutoDock Tools and exported in PDBQT format for docking analysis.

### 2.9 Docking Analysis and Visualization of Binding Conformations

AutoDockVina was employed to conduct the docking analysis. AutoDockVina performs an iterated local search algorithm using the coordinates of the search space that is provided by the user. The synthetic derivatives were designed using ChemDraw Ultra 12.0; their three‐dimensional structures, as well as those of the target proteins, were obtained with the aid of various bioinformatics software, including Discovery Studio v16.1.0.15350, AutoDock Tools v1.5.6, and PyRx v0.8. In this work, molecular docking was conducted to examine the interactions between these synthetic derivatives and H^+^/K^+^ ATPase with PDB ID: 5YLU and Cyclooxygenase‐II with PDB ID: 3LN1, comparing their binding affinities.

### 2.10 Docking Protocol Validation by Re‐Docking

Validation of molecular docking methodology was conducted based on the re‐docking technique, using the co‐crystallized reference ligands, which are part of the selected protein structures. Celecoxib and omeprazole served as validation ligands for COX‐2 (PDB ID: 3LN1) and H^+^/K^+^‐ATPase (PDB ID: 5YLU), correspondingly. Before being re‐docked, the crystallographic ligands were dissociated from their respective protein structures, and preparation of protein structures was performed in the same manner as receptor preparation for the subsequent docking experiments. Crystallographic coordinates of reference ligands were preserved as reference binding poses. Then, the obtained reference ligands were re‐docked into the corresponding experimentally identified binding sites using the same molecular docking program, parameters of receptor preparation, coordinates and sizes of grid, and docking parameters as in the case of synthesized benzimidazole–pyrazole derivatives. Such an approach was chosen in order to replicate the real docking conditions for the test compounds and reference drugs. Several binding poses were generated for each ligand, and the best‐ranked pose was chosen in terms of docking score and compared to the experimental crystallographic pose.

### 2.11 MD Simulation

A 100‐ns molecular dynamics simulation was performed for the structural stability and dynamic behavior of protein‐ligand complexes using the GROMACS 2025.1 package. The simulation was performed in isothermal and isobaric (NPT) ensemble at 300 K and 1 atm. The time‐dependent parameters discussed for the analysis of stability and conformational behavior of these complexes are root mean square deviation (RMSD), root mean square fluctuation (RMSF), radius of gyration (Rg), and intermolecular hydrogen bonds. The analysis of the MD simulation will help to demonstrate the microscopic stability of the protein and protein‐ligand complex. It will estimate the structure and conformational behavior of the complex. Proteins were simulated using the GROMACS 2025.1 package [27]. The simulation was performed using the CHARMM General Force Field for proteins. Topologies for the ligands were prepared using the SwissParam server. In these simulation studies, energy minimization was performed in a vacuum using 2500 steps of the steepest descent algorithm to remove steric clashes. The simulation was performed using the simple point charge (SPC) model for simulating water. Na^+^ and Cl^−^ ions were introduced as counterions using the gmx genion tool of the GROMACS package. After this, the equilibrated system underwent two‐step stabilization: an initial 100 ps of NVT equilibration, which keeps the number of particles, volume, and temperature constant, with gradual heating up to 300 K. This is followed by the second step of equilibration of pressure, temperature, and density for 100 ps. All these steps constrained all covalent bonds via the LINCS algorithm; regulated the system parameters via V‐rescale thermostat and Parrinello‐Rahman barostat; and calculated the long‐range electrostatic interactions using the particle mesh Ewald method. After equilibration, a 100 ns production MD simulation was performed for each system under periodic boundary conditions to capture the dynamic behavior of protein‐ligand complex.

### 2.12 Root Mean Square Deviation (RMSD)

RMSD was used to quantify the average positional deviation of atoms between trajectory frames during the simulation. RMSD for a given frame *x* was calculated as:


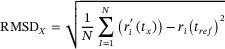




The selected atom set contained a total of nitrogen atoms. The reference time (t_ref_​) was set to the first frame (*t* = 0). For each frame *x*, with capture time t_x_​, the positions of the selected atoms after alignment to the reference structure were denoted as r_i_′​(t_x_​). Alignment was performed to eliminate overall translational and rotational motions, ensuring that the calculated deviations reflected only internal conformational changes. RMSD calculations were performed for all frames in the trajectory, and the resulting profiles were analyzed and visualized using XMGrace [28].

#### 2.12.1 Root Mean Square Fluctuation (RMSF)

RMSF was calculated to determine the local flexibility of residues based on their positional deviations during the molecular dynamics simulation. RMSF measures the average positional fluctuation of each residue relative to a reference structure throughout the simulation, providing insight into the flexible regions within the protein chain. The RMSF for residue *i* was computed as:

$$\;\mathrm{RMS}F_{I}=\sqrt{\frac{1}{T}\sum_{I=1}^{T}\left(r_{i}^{\prime }\left(t\right)\right)-r_{i}{\left(t_{ref}\right)}^{2}}$$

where *T* is the total number of frames, r_i_′(t) is the position of residue *i* at time *t* after superposition on the reference structure, and r_i_(t_ref_) is its position in the reference frame, typically the first frame (*t* = 0). The angle brackets show averaging over the selected atoms of each residue. Peaks in the RMSF profile represent regions of higher structural flexibility, typically corresponding to loop regions and the N‐ and C‐terminal ends of the protein, whereas core residues generally display lower fluctuations [29].

#### 2.12.2 Radius of Gyration (Rg)

The Rg was calculated to test the compactness of the protein structure during the simulation. Rg is defined as the mass‐weighted root mean square distance of the atoms from their common center of mass and provides insight into overall structural stability and folding characteristics over time [30]. Lower fluctuations in Rg indicate maintenance of structural compactness, whereas significant changes suggest unfolding or conformational expansion.

#### 2.12.3 Solvent Accessible Surface Area (SASA)

The calculation of the Solvent Accessible Surface Area (SASA) was performed using the gmx SASA module within GROMACS version 2025.1 for a period of 100 ns. These calculations were performed on the whole protein‐ligand complex with a probe radius set to the default value of 0.14 nm and standard parameters for the solvent properties, and these SASA results were obtained at regular intervals to note changes associated with solvent accessibility, giving information about stability and dynamics related to folding [31].

#### 2.12.4 Hydrogen Bonds

Intra‐molecular hydrogen bonding was studied employing the gmx hbond command in GROMACS v2025.1 over a period of 100 ns. Conventional geometric criteria were used, with a donor‐acceptor distance threshold cut‐off value set to ≤0.35 nm and a hydrogen‐donor‐acceptor bond angle set to ≥120°. The number of hydrogen bonds was calculated for each frame to examine system stability and integrity [32].

### 2.13 MMGBSA Binding Free Energy

The binding free energy (ΔG_bind_) of the ligand–protein complex was estimated using the Molecular Mechanics/Generalized Born Surface Area (MM/GBSA) approach implemented in *gmx_MMPBSA* v1.6.3, which merges GROMACS with AmberTools for end‐state free energy calculations. The total binding free energy was decomposed into van der Waals, electrostatic, and solvation contributions according to:

$$\Delta \;G_{bind}=\;\Delta E_{vdW}+\;\Delta E_{ele}+\;\Delta G_{polar}+\;\Delta G_{polar}$$

The polar solvation energy was calculated with the GB implicit solvent model using igb = 5 setting corresponding to GB‐Neck2, while the nonpolar contribution was obtained from the solvent‐accessible surface area using the LCPO method. Entropic contributions were neglected, following the standard protocols adopted in the estimation of relative binding affinity [33]. With a total time length of 100 ns, 1001 evenly spaced snapshots were extracted from the production trajectory of the protein–ligand complex. The binding free energies were then calculated for the complex, isolated receptor, and ligand; the final binding energy obtained from their comparative evaluation:

$$\Delta \;G_{bind}=G_{complex}-\left(G_{receptor}+G_{ligand}\right)$$

In this study, we utilized MD simulations in cases where the AlphaFold prediction and the subsequent validation tools indicated acceptable quality and structural integrity [34].

### 2.14 In Vivo Pharmacological Evaluation of Benzimidazole‐Pyrazole Hybrids

#### 2.14.1 Evaluation of Anti‐Ulcer Activity

Anti‐ulcer property of a series of derivatives to prevent ethanol‐associated gastric ulcers in adult albino rats was investigated. To this end, healthy animals (180 to 220 g) of either male or female were appointed to experimental groups consisting of six rats each. The participants in all groups remained without food 24 h before the experiment with unlimited admittance to water. The ulcer model was induced by oral intake of 96 % ethanol dose of 5 mL/kg. The control group ended up getting normal saline (5 mL/kg) instead. Test derivatives (**5a, 5b, 5e, 5g,** and **5i**) were used at a dosage of 10 mg/kg in the treatment groups. The positive control was omeprazole (30 mg/kg).

The rats were pinned an hour after therapy, and the stomachs were again observed to test the gastric ulceration. All measurements were restricted to the glandular area, where the lesions were observed and their area of lesion measured. On these measurements, an animal was awarded an ulcer index (UI) based on the following scoring system. No dose related adverse effects were identified. Relative to the control cohort, several test compounds triggered pronounced decreases in the mean UI: **5a** (*p* = 0.001), **5b** (*p* = 0.004), **5e** (*p* = 0.052), **5g** (*p* = 0.001), and **5i** (*p* = 0.045). These compounds had efficacies that were comparable to those of omeprazole.

0 = no ulcer, 1 = ulcer surface area (US) ≤ 0.5 mm^2^, 2 = 0.5< US ≤ 2.5 mm^2^, 3 = 2.5< US ≤ 5 mm^2^, 4 = 5< US ≤ 10 mm^2^, 5 = 10< US ≤ 15 mm^2^, 6 = 15< US ≤ 20 mm^2^, 7 = 20< US ≤ 25 mm^2^, 8 = 25< US ≤ 30 mm^2^, 9 = 30< US ≤ 35 mm^2^, 10 = US > 35 mm^2^

The ulcer index was determined by summing the lengths (in mm) of all wounds observed in each stomach. The protective effect of the evaluated derivatives was stated as the percentage of ulcer inhibition using the formula:

$$I\left(\%\right)=\left(USc-USt\right)\times 100/USc$$

where, USc = ulcer surface area of control and USt = ulcer surface area of test animal [35].

#### 2.14.2 Inhibition of Gastric H^+^/K^+^ ATPase Activity

A series of five synthesized derivatives (**5a, 5b, 5e, 5 g,** and **5i**) were studied with respect to the in vitro inhibition of the gastric H^+^/K^+^ ATPase enzyme using a colorimetric assay procedure as reported [36]. The assay is based on the measure of inorganic phosphate (Pi) released through hydrolysis of adenosine triphosphate, so it measures enzyme activity. The gastric mucosal tissue of rats was homogenized, and the homogenate was centrifuged at 3500 rpm, 10–15 min to collect the supernatant. Spectrophotometric determination of Pi release was done in the supernatant at 660 nm. Omeprazole was taken as a comparative standard. ATPase activity was stated as 1 mmol Pi released per milligram of tissue protein/hour/unit (anaerobic ATPase activity at pH 9.0: 1 unit = 1 unit of enzyme hydrolyzing 1–103 Pi/1 mg protein/hour). The inhibition potentials of each compound were computed and reported appropriately.

#### 2.14.3 Acute Oral Toxicity Assessment

They assay was carried out as stipulated by the OECD [37]. 12 healthy adult albino rats (Wistar strain) of around 250 ± 10 g weight were obtained in the animal house of the Department of Pharmacy, Riphah Institute of Pharmaceutical Sciences. The animals were arbitrarily assigned four treatment groups of three rats each. The control group (Group A) was injected with normal saline and Group B, C, and D were injected with drug **5a, 5e,** and **5i** respectively that were chosen based on their attractive pharmacological potential. Animals were kept in a regular laboratory environment (temperature 25°C ± 2°C, relative humidity 65% ± 5%, 12 h light/dark cycle) with free food and water. Oral administration of the test compounds at a maximum tolerable dose (MTD) of 100 mg/kg body weight was carried out. In control animals 0.9 % saline solution was used at 1 mL/100 g body weight. During the 14 days of observation subjects were observed daily to report side effects manifesting through fur, skin, eyes, and mucous membranes, respiratory and cardiovascular disorders, autonomic and central nervous system, behavioral patterns, locomotor activity, tremor, convulsions, lethargy, salivation, sleep disturbance, and coma. Prior to dosing and on regular intervals during study, body weights were recorded.

#### 2.14.4 Indomethacin‐Induced Gastric Ulcer Model

In order to assess the gastro protective effects of the structured analogues, a modified indomethacin‐induced gastric ulcer model of Djahanguri was used [38]. Rats kept overnight without food were orally given vehicle (2% Tween 80), designated series of test compounds (**5a, 5b, 5e, 5g,** and **5i)** at predetermined doses, or a reference ant‐ulcer agent. 50 mg/kg indomethacin in 2% sodium bicarbonate was administered with one dose to induce ulcers after 1 h of treatment. The animals were euthanized using cervical dislocation after 4 h and the stomachs dissected out. Infusion of a 1 mL of 5% formaldehyde was performed to outline ulcer regions. The stomachs were then opened along the greater curvature, their contents were emptied, and the mucosa rinsed gently with saline. These samples were stored and used in biochemical tests.

#### 2.14.5 In vivo Antioxidant Activity Analysis

The in vivo antioxidant potential of the synthesized compounds was evaluated using several biochemical markers in the gastric mucosal tissue of rats.

#### 2.14.6 Myeloperoxidase (MPO) Activity

In indomethacin‐induced gastric ulceration, a surrogate method of quantifying the activity of MPO was used as a marker of the presence of neutrophil infiltrates and inflammation [39]. An aliquot of gastric tissue homogenate was added to 290 µL of the phosphate‐buffered saline solution with o‐dianisidine dihydrochloride (0.167 g/L) and 0.005% hydrogen peroxide. Absorbance was read at 460 nm after 10 min of 37°C incubation. The findings were presented in the form of units per gram of tissue (U/g tissue).

#### 2.14.7 Malondialdehyde (MDA) Levels

The method of Ohkawa et al. was used to determine the morphologically distinct adducts derived by the process of lipid peroxidation in the gastric tissue [40]. The recently removed gastric contents were carefully cleaned using 0.9% saline, dried and weighed down before being homogenized using 0.15 M KCl. The homogenate was subsequently agitated with 1.5 mL of 20% acetic acid and 2.0 mL of 20% sodium dodecyl sulfate (SDS). The pH of the solution was brought to 3.5 using NaOH and the mixture was allowed to react at 95°C in a water bath for 1 h. The suspensions were subsequently centrifuged at 2600 g, 10 min following a short period of cooling. The supernatant absorbance was taken at 532 nm and MDA concentration was presented as millimoles per gram of tissue.

#### 2.14.8 Prostaglandin E2 (PGE2) Quantification

The protective effect of different compounds against gastric mucosa was determined through the endogenous prostaglandin pathway based on the level of PGE2. Gastrointestinal tissue was homogenized and frozen to a low −80°C until analysis. To measure PGE2 concentrations, a commercially produced ELISA kit (Stressgen, USA) was used following the manufacturer instructions.

#### 2.14.9 Tumor Necrosis Factor‐Alpha (TNF‐α) Levels

Anti‐inflammatory activity of evaluated derivatives was determined by plasma TNF‐alpha levels in rats with the IND‐ulcer model. Indomethacin was used to induce ulcers pre‐treated with the respective compounds of the test or vehicle. After 4 h, the anesthetic produced by sodium thiopental (50 mg/kg, intraperitoneal) and chloral hydrate (300 mg/kg, intraperitoneal) was administered, and blood was sampled via the inferior vena cava in EDTA tubes. This was centrifuged at 3500 rpm; 3500 rpm speed, 10 min, 4°C (T) and kept at −80°C till the time of running the analysis. Hemolyzed samples were omitted. The levels of TNF‐alpha were determined by ELISA kits (eBioscience, USA and Immuno Biological Laboratories, Japan) following the instructions of the different manufacturers.

#### 2.14.10 Statistical Analysis

Values are expressed as means ± SEM. Statistical analyses were conducted with the help of GraphPad Prism 8 software. Data distribution was assessed using the Shapiro–Wilk test, and homogeneity of variance was assessed using Levene's test. Multiple groups comparison tests were performed with the help of one‐way ANOVA followed by Tukey's multiple comparisons test.

## 3 Results

### 3.1 Docking Studies

Docking results of synthesized benzimidazole‐pyrazole hybrids against H^+^/K^+^ ATPase (PDB ID: 5YLU) and Cyclooxygenase II (PDB ID: 3LN1) demonstrated that compounds make a stable complex and suitable binding affinity as compared to standard drug omeprazole and celecoxib (Table 1). Two‐dimensional pattern (2D) of these compounds with target protein shown that hydrogen of amino group of imidazole ring interacted through hydrogen bonding with different amino acids. Pi sigma, pi‐alkyl and pi‐donor hydrogen bond interactions were seen between the aromatic rings of derivatives and different amino acid residues of active site of target protein.

**TABLE 1:** Binding affinities (Kcal/mol) (**Δ**G) of ligands **(5a–5j)** with target proteins.

Compound	Highest binding affinity (Kcal/mol) (ΔG)			
	COX‐2 (3LN1)	Amino Acid Interactions	H^+^/K^+^ ATPase (5YLU)	Amino Acid Interactions
**5a**	−8.3		−8.7	
**5b**	−8.4		−9.0	
**5c**	−8.5		−7.9	
**5d**	−8.9		−8.9	
**5e**	**−9.3**	TYR 324, LEU 321, 328, TRP 356, ALA 496, VAL 318, 492 SER 322	−8.9	
**5f**	−8.3		−9.0	
**5g**	−8.5		−8.2	
**5h**	−8.1		−8.6	
**5i**	−9.1		−**9.4**	GLU 64, 249, ILE 16, 306
**5j**	−9.0		−8.7	
**Celecoxib**	**−9.4**	TYR 324, 354, LEU 321, 328, 353, TRP 324, 356, ARG 89, ALA 496, GLN 161, VAL 318, 492, SER 322	—	
**Omeprazole**	**—**		**−8.2**	ARG 288, 356, ILE 16, 17, 306, LEU 298, GLU 64, 249

**FIGURE 6:**
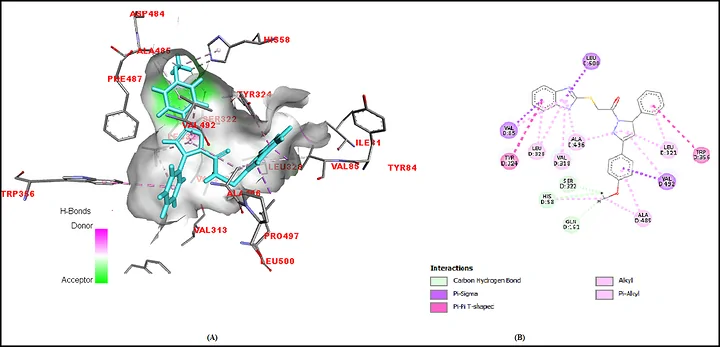
Binding interactions of compound 5e with the COX‐2 active site (PDB ID: 3LN1) shown in 3D (A) and 2D (B) representations.

**FIGURE 7:**
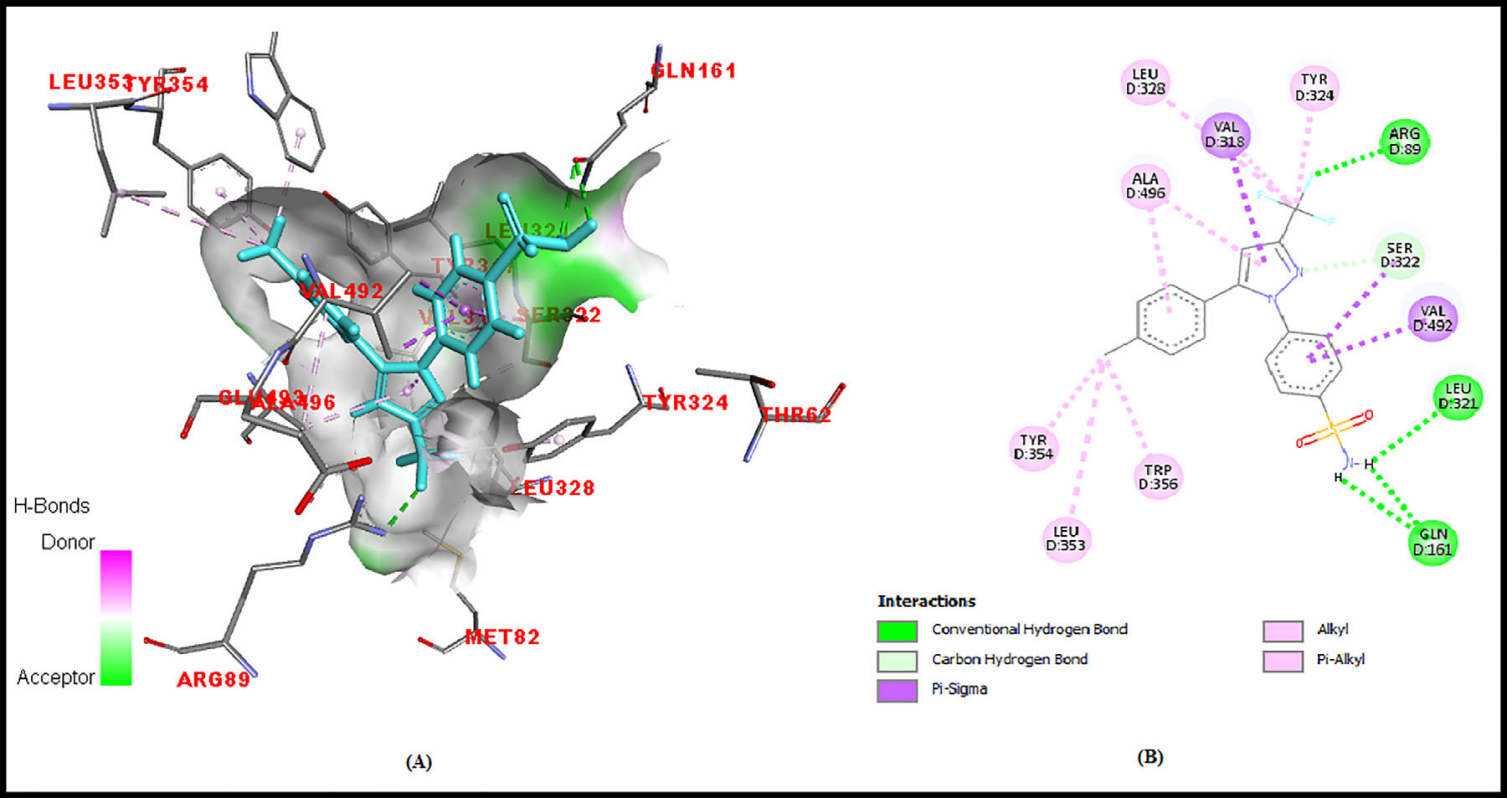
Binding interactions of Celecoxib with the COX‐2 active site (PDB ID: 3LN1) shown in three‐dimensional (A) and two‐dimensional (B) representations.

**FIGURE 8:**
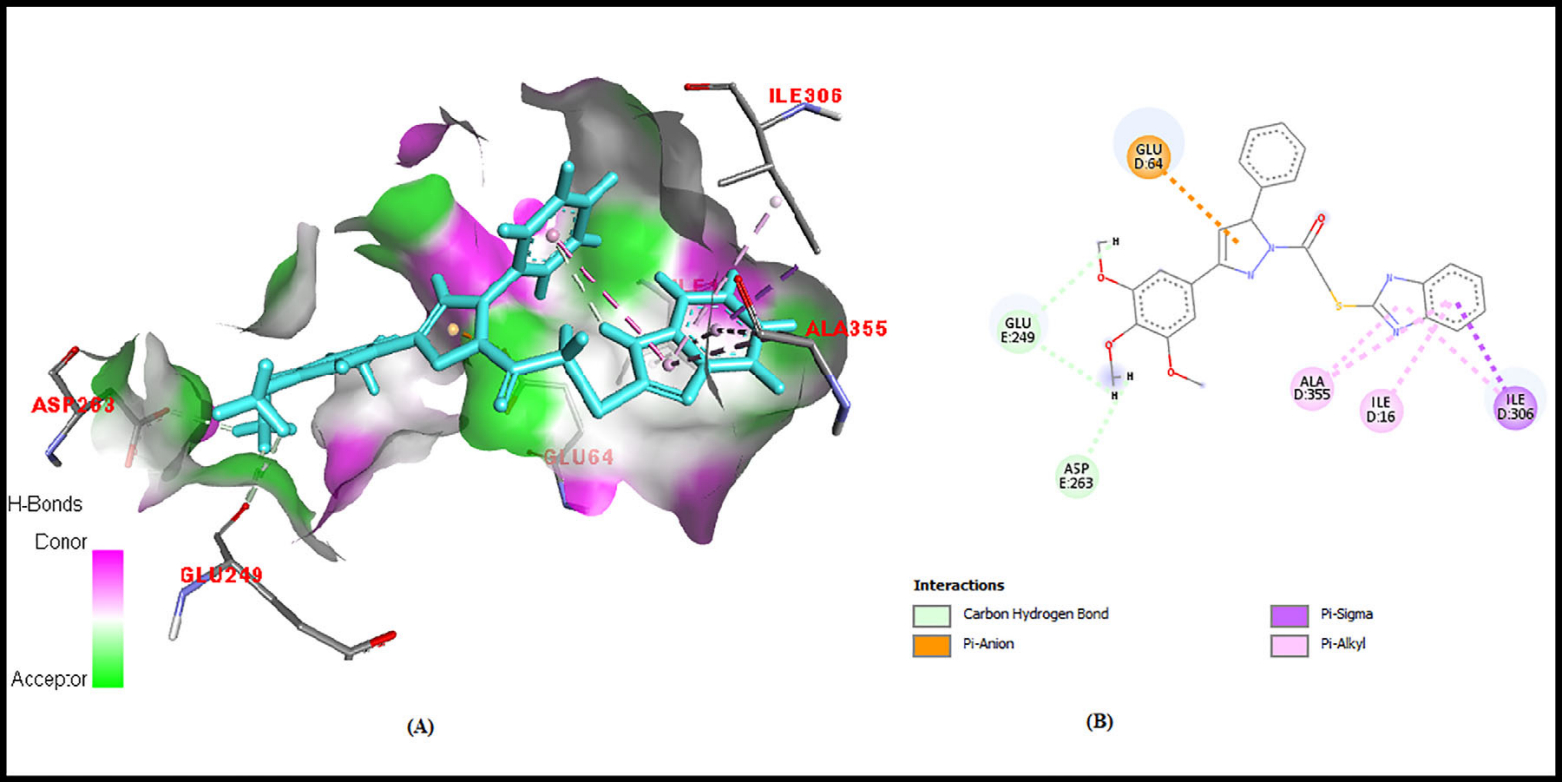
Binding interactions of compound 5i with the H^+^/K^+^ ATPase active site (PDB ID: 5YLU) shown in three‐dimensional (A) and two‐dimensional (B) representations.

**FIGURE 9:**
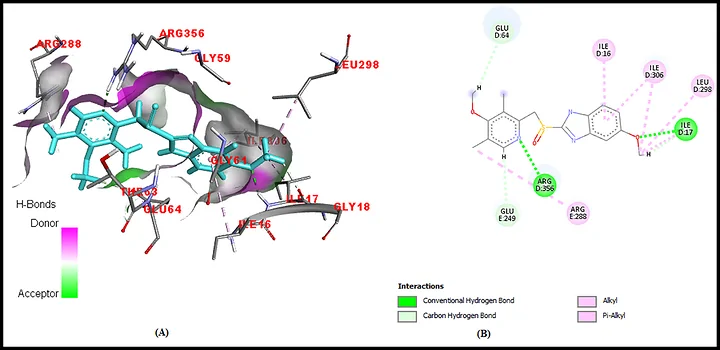
Binding interactions of Omeprazole with the H^+^/K^+^ ATPase active site (PDB ID: 5YLU) shown in three‐dimensional (A) and two‐dimensional (B) representations.

The synthesized compounds were investigated through docking against H^+^/K^+^ ATPase (PDB ID: 5YLU), and COX‐2 (PDB ID: 3LN1) and it was noted that some derivatives showed desirable binding affinities. Compound 5e showed best interaction with COX‐2 with a binding energy of −9.3 kcal/mol (Figure 6). Interaction of Celecoxib with COX‐2 showing binding energy of −9.4 kcal/mol (Figure 7). Interaction of compound 5i with H^+^/K^+^ ATPase binding site (PDB ID: 5YLU) with a binding energy of −9.4 kcal/mol (Figure 8). Interaction of Omeprazole with H^+^/K^+^ ATPase showing binding energy of −8.2 kcal/mol (Figure 9). Docking data of synthesized compounds support the inhibitory effects of all the tested compounds but among these 5e showed best results against COX‐2, while 5i showed promising results against H^+^/K^+^ ATPase.

### 3.2 Docking of 5i and Omeprazole With H^+^/K^+^ ATPase (PDB ID: 5YLU)

Amino acids present in H^+^/K^+^ ATPase include ARG 288, 356, ILE 16, 17, 306, LEU 298, GLU 64, 249 which interact with omeprazole and amino acids GLU 64, 249, ILE 16, 306 show interaction with 5i, this result shows that omeprazole and 5i shows some common amino acid interactions.

### 3.3 Docking of 5e and Celecoxib with COX‐2 (PDB ID: 3LN1)

Amino acids present in COX‐2 include TYR 324, 354, LEU 321, 328, 353, TRP 324, 356, ARG 89, ALA 496, GLN 161, VAL 318, 492, SER 322 which interact with celecoxib and amino acids TYR 324, LEU 321, 328, TRP 356, ALA 496, VAL 318, 492, SER 322 show interaction with 5e, this result shows that celecoxib and 5e shows some common amino acid interactions.

### 3.4 RMSD Calculation

**FIGURE 10:**
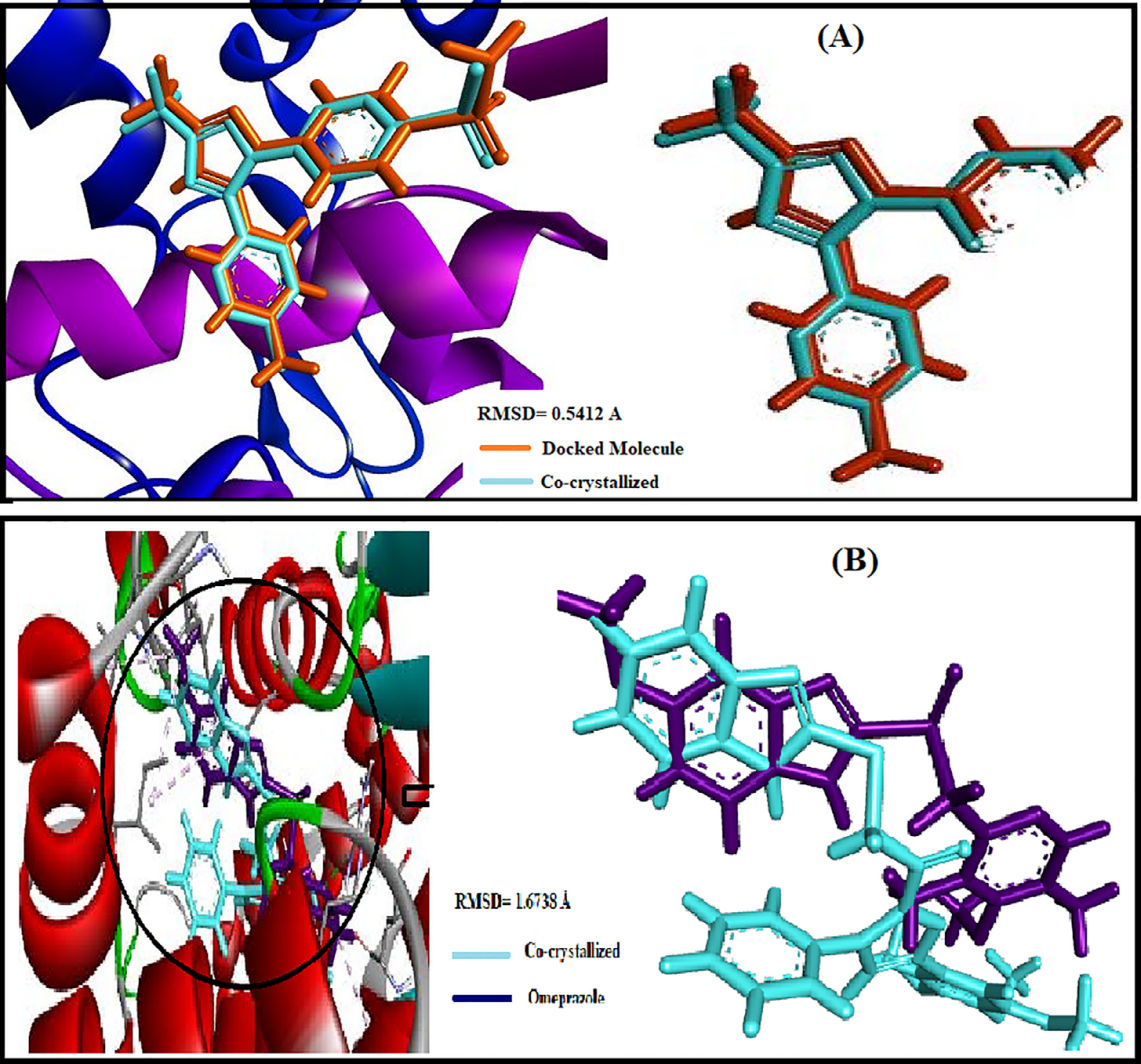
(A) RMSD value for celecoxib against co‐crystallized celecoxib was 0.5412 Å. (B) RMSD value for omeprazole against co‐crystallized omeprazole was 1.6738 Å.

The 3D complexes showed very similar conformation to that of the co‐crystallized complexes. In the study, re‐docking of the ligands to the protein targets was carried out. The RMSD value of compound 5i to omeprazole was 1.6738 Å, while that of compound 5e to celecoxib was 0.5412 Å (Figure 10A,B).

### 3.5 MD Simulation of Selected Proteins with Ligand (5e)

#### 3.5.1 Root Mean Square Deviation (RMSD)

**FIGURE 11:**
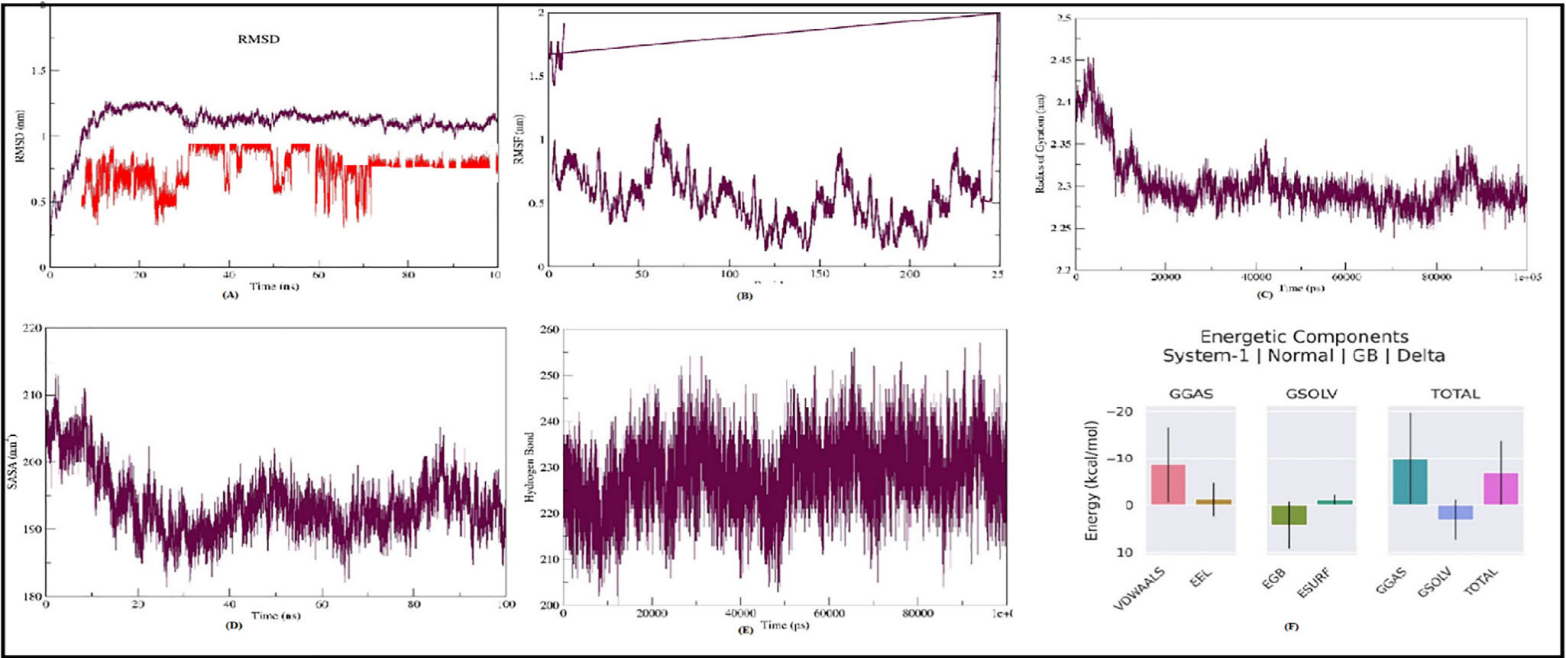
(A) RMSD of 3LN1 (magenta) and 5e (red) (B) RMSF of 3LN1 (C) Radius of Gyration of the protein‐ligand complex (D) Solvent accessible surface area of the protein‐ligand complex (E) Hydrogen bonds between the protein and ligand (F) MM/GBSA binding free energy components.

**FIGURE 12:**
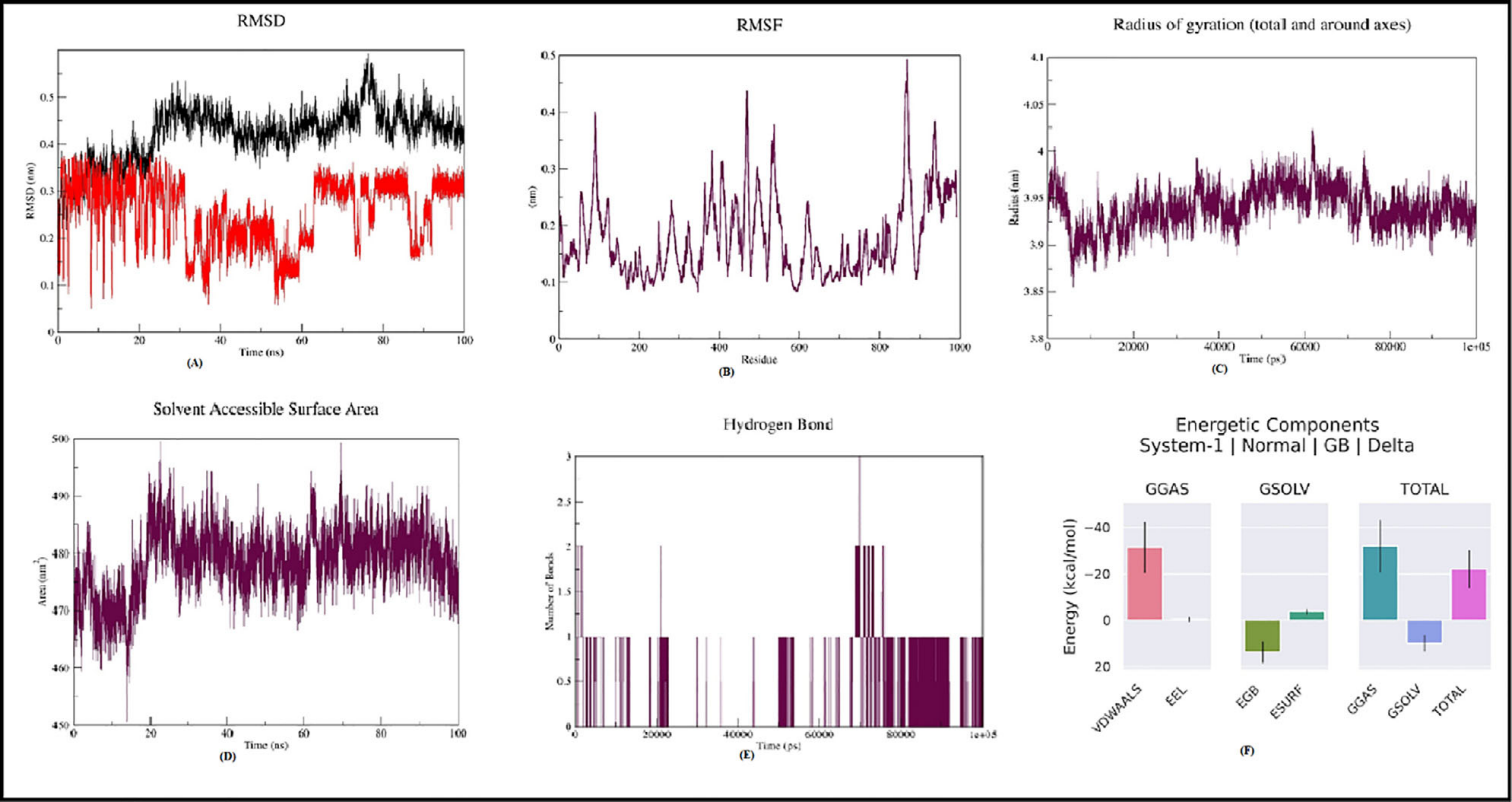
(A) RMSD of 5YLU (black) and 5e (red) (B) RMSF of 5YLU (C) Radius of Gyration of the protein‐ligand complex (D) Solvent accessible surface area of the protein‐ligand complex (E) Hydrogen bonds between the protein and ligand (F) MM/GBSA binding free energy components.

The RMSD profiles for the protein (3LN1) backbone (magenta) and ligand (red) over 100 ns are shown in Figure 11A. The protein RMSD began at ∼0.35 nm, increased gradually to ∼1.25 nm by 18 ns, and then remained stable with minor fluctuations, indicating structural equilibration. The RMSD profiles for the protein (5YLU) backbone (black) and ligand (red) over 100 ns are shown in Figure 12A. The protein RMSD began at ∼0.30 nm, increased gradually to ∼0.52 nm by 50 ns, and then remained stable with minor fluctuations, indicating structural equilibration. The ligand RMSD was consistently lower, fluctuating between 0.13–0.35 nm, with brief drops below 0.15 nm, suggesting strong positional stability within the binding pocket. Convergence of both RMSD curves after ∼20 ns indicates that the system attained equilibrium early in the simulation.

#### 3.5.2 Root Mean Square Fluctuation (RMSF)

The RMSF profile of 3LN1 describes the per‐residue flexibility of the protein, with a majority of the residues having low fluctuations around ∼0.65–1.15 nm, reflecting structurally stable regions. Distinct peaks reaching ∼1.10–1.20 nm are seen in certain loop and terminal segments, reflecting the higher mobility characteristic of these regions (Figure 11B). The RMSF profile of 5YLU describes the per‐residue flexibility of the protein, with the majority of the residues exhibiting low fluctuations around ∼0.15–0.28 nm, reflecting structurally stable regions. Distinct peaks reaching ∼0.42–0.50 nm in certain loop and terminal segments reflect the higher mobility characteristic of these regions. In contrast, residues in the protein core have low RMSF values, reflecting structural rigidity and supporting the stability of the ligand‐binding region (Figure 12B).

### 3.6 Radius of Gyration (Rg)

The Rg profile of 3LN1 shows a fluctuation in the range of 2.20 to 2.50 nm along the 100 ns trajectory, starting from ∼2.38 nm and subsequently undergoing a minor reduction to ∼2.15 nm in the early phase. This indicates minor compaction of the structure after equilibration. From then on, Rg remains stable at an average of 2.12 ± 0.02 nm, reflecting preservation of overall structural compactness (Figure 11C). The Rg profile of 5YLU depicts a fluctuation in the range of 3.85 to 4.02 nm along the 100 ns trajectory, starting from ∼3.90 nm and subsequently reduced to ∼3.85 nm in the early phase, which indicates minor compaction of the structure after equilibration. From then on, Rg remains stable at 3.83 ± 0.02 nm, reflecting preservation of overall structural compactness (Figure 12C).

### 3.7 Solvent Accessible Surface Area (SASA)

For the SASA profile, 3LN1 fluctuates from 184 to 210 nm^2^, which rises from ∼191 to ∼209 nm^2^ during the first 20 ns, indicating the initial structural relaxation and slight expansion of the protein surface. After this adjustment, SASA stabilizes around an average value of ∼196 nm^2^ for the rest of the simulation, reflecting a consistent solvent exposure pattern (Figure 11D). For the SASA profile, 5YLU fluctuates between 465 and 498 nm^2^, with a rising trend in the first 20 ns from ∼465 to ∼475 nm^2^, reflecting the initial structural relaxation and slight expansion of the protein surface. After this adjustment, SASA stabilizes around an average value of ∼479 nm^2^ for the rest of the simulation, reflecting a consistent solvent exposure pattern (Figure 12D).

### 3.8 Hydrogen Bonds

The hydrogen bond profile of 3LN1 indicates intermittent formation of H‐bonds between the ligand and the protein, with counts ranging from 0 to 2.2 throughout the 100 ns simulation. For the majority of the trajectory, the occupancy is between 0–2 bonds, with brief periods from 40–60 ns where 2–3 bonds are present (Figure 11E). The hydrogen bonding profile of 5YLU shows intermittent formation of H‐bonds between the ligand and protein, with counts ranging from 0 to 3 throughout the 100 ns simulations. In the majority of the trajectory, the occupancy is at 0–1 bond, with short periods from 60–80 ns showing 2–3 bonds (Figure 12E).

### 3.9 MMGBSA

The MM/GBSA binding free energy of both proteins is presented in Table 2. The calculated MM/GBSA (Molecular Mechanics Generalized Born Surface Area) binding free energy (3LN1) gave a total ΔGbind of –20.46 kcal/mol, showing that the protein–ligand interaction was favorable and stable. Thermodynamically, van der Waals forces (ΔEvdW = −34.17 kcal/mol) constituted the most important stabilizing interactions, reflecting the dominant role played by hydrophobic interactions in the stabilization of this complex. Electrostatic interactions contributed insignificantly (ΔEele = −0.54 kcal/mol), which reflected minimal direct charge–charge interactions. Polar solvation energy was destabilizing (ΔGpolar = +14.27 kcal/mol), which stood for the energetic cost of desolvating polar groups. While polar solvation was unfavorable, non‐polar solvation gave extra stability (ΔGnonpolar = −3.17 kcal/mol), in agreement with hydrophobic surface burial upon ligand binding (Figure 11F). The gas‐phase interaction energy totaled −28.23 kcal/mol, while the solvation free energy was slightly destabilizing at +10.33 kcal/mol. MM/GBSA binding free energy calculation of 5YLU produced the total ΔGbind value of −22.22 kcal/mol, suggesting that protein–ligand complexation is thermodynamically favorable. As summarized in Table 2, van der Waals forces (ΔE_vdW_​ = −31.48 kcal/mol) were the dominant stabilizing factor, highlighting the key role of hydrophobic interactions in complex stabilization. Electrostatic contributions were minimal (ΔE_ele_​ = −0.52 kcal/mol), suggesting limited direct charge–charge interactions. Polar solvation energy was destabilizing (Δ_Gpolar_ = +13.53 kcal/mol), reflecting the energetic cost of desolvating polar groups. In contrast, nonpolar solvation provided additional stabilization (ΔG_nonpolar_ = −3.77 kcal/mol), consistent with hydrophobic surface burial upon ligand binding. The gas‐phase interaction energy totaled −31.91 kcal/mol, whereas the solvation free energy was slightly destabilizing at +9.88 kcal/mol (Figure 12F). Overall, the large negative total binding energy confirms a stable complex mainly influenced by van der Waals forces, with nonpolar solvation making a supportive contribution.

**TABLE 2:** MM/GBSA binding free energy components for the protein–ligand complex.

Energy component	Value (kcal/mol) (5YLU)	Value (kcal/mol) (3LN1)
VDWAALS	−31.48	−34.17
EEL	−0.52	−0.54
EGB	13.53	14.27
ESURF	−3.77	−3.17
GGAS	−31.91	−28.23
GSOLV	9.88	10.33
Total ΔG	−22.22	−20.46

### 3.10 In Vivo Pharmacological Evaluation of Benzimidazole‐Pyrazole Hybrids

#### 3.10.1 Anti‐Ulcer Activity (Ethanol Induced Model)

**FIGURE 13:**
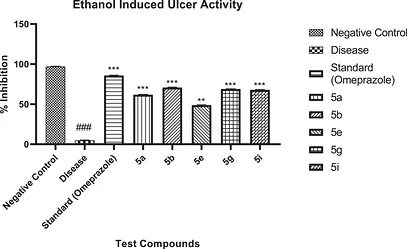
Anti‐ulcer effects of benzimidazole‐pyrazole hybrids and omeprazole in rats. Data are mean ± SEM (*n* = 6/group). *p < 0.05, **p < 0.01, ***p < 0.001 versus omeprazole; #*p* < 0.05 versus ulcer control. Analysis by one‐way ANOVA followed by Tukey's test (GraphPad Prism 8).

**FIGURE 14:**
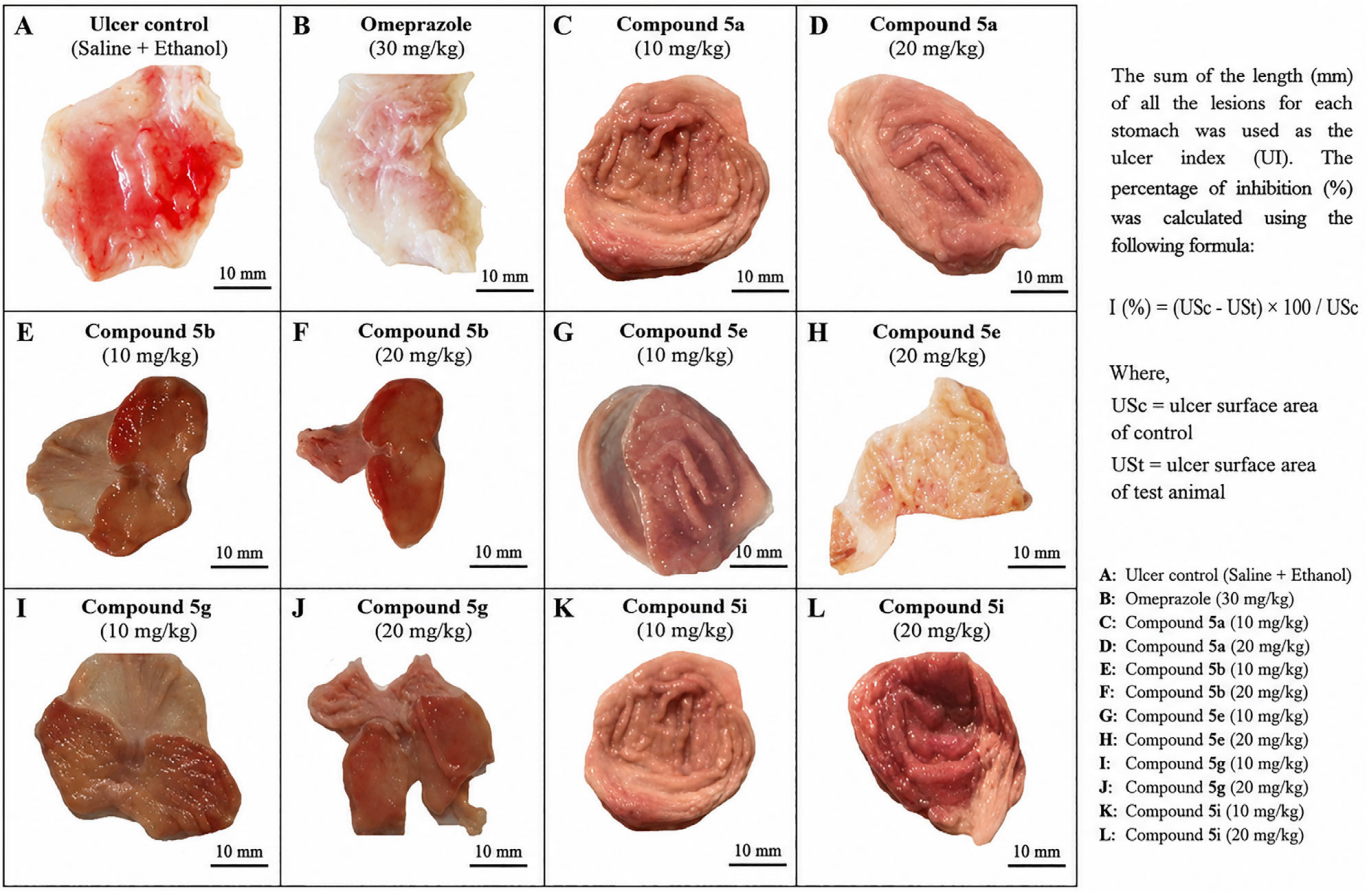
Gross‐appearance of rats’ gastric mucosa in saline and pre‐treated groups. (A) Pre‐treated with normal saline (5 mL/kg), (ulcer control group), critical injuries are seen, as administration of 96% ethyl alcohol (5 mL/kg) overproduced hemorrhagic necrosis of gastric mucosa, (B) pre‐treated with omeprazole, 30 mg/kg, (C–L) pre‐treated with compound **5a**, **5b**, **5e**, **5** **g**, and **5i**, at dose of 10 and 20 mg/kg respectively.

Most of the newly synthesized benzimidazole–pyrazole hybrids demonstrated notable anti‐ulcer activity. Omeprazole, used as the reference drug, exhibited an inhibition rate of 85% ± 4%. Among the tested compounds, derivatives 5b, 5 g, and 5i showed strong anti‐ulcer effects (***p < 0.001), reducing ulceration by 70% ± 4.5%, 68.2% ± 5.4%, and 67.1% ± 5.7%, respectively. In contrast, compounds 5a and 5e displayed moderate activity, with ulcer inhibition rates of 61% ± 3.3% and 48.8% ± 3.8%, respectively. The histological assessment of gastric mucosa indicated a marked reduction in lesions in pre‐treated groups compared to the ulcer control group, as illustrated in (Figures 13 and 14).

### 3.11 Inhibition of Gastric H^+^/K^+^ ATPase Activity

The blocking of gastric Hydrogen‐potassium ATPase activity was conducted for a subset of the synthesized benzimidazole‐pyrazole hybrids **(5a, 5b, 5e, 5 g,** and **5i)** using a commercially available colorimetric assay kit. The enzymatic activity was expressed in terms of µmol Pi/mg protein/h. The standard reference drug omeprazole was included for comparative analysis. Experimental procedures were performed in triplicate (*n* = 3) to ensure reliability.

**FIGURE 15:**
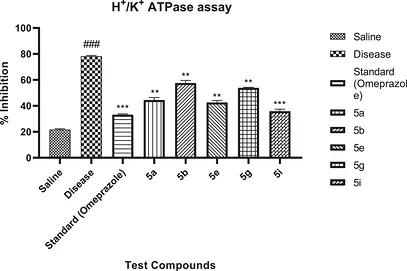
Effect of benzimidazole‐pyrazole hybrids (5a, 5b, 5e, 5 g, and 5i) and omeprazole in rat gastric H^+^/K^+^ ATPase assay. Data are mean ± SEM (*n* = 3/group). *p < 0.05, **p < 0.01, ***p < 0.001 versus negative control (Ulcerative); #*p* < 0.05 versus saline. Analysis by one‐way ANOVA followed by Tukey's test (GraphPad Prism 8).

The results, revealed that compound **5i** demonstrated superior inhibitory activity among the tested compounds, with a value of 35.81 ± 2.91 µmol Pi/mg protein/h, which is marginally higher than that of omeprazole (33.18 ± 2.89 µmol Pi/mg protein/h). Notably, compound **5e** also exhibited better activity, recording a value of 42.51 ± 2.77 µmol Pi/mg protein/h, indicating a significant suppression of proton pump activity. These findings suggest that selected derivatives, particularly **5e** and **5i**, possess strong inhibitory potential against gastric proton pumps and may serve as promising leads for anti‐ulcer drug development (Figure 15).

### 3.12 Indomethacin‐Induced Gastric Ulcer (IND‐Ulcer) Model

**FIGURE 16:**
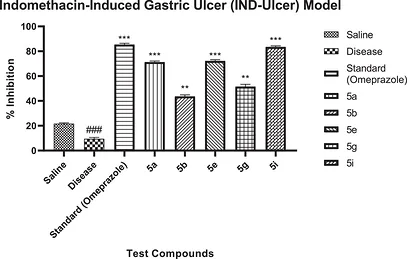
Anti‐ulcer effects of benzimidazole‐pyrazole hybrid compounds and omeprazole in rats. Data are mean ± SEM (*n* = 6/group). *p < 0.05, **p < 0.01, ***p < 0.001 versus omeprazole; #*p* < 0.05 versus ulcer control. Analysis by one‐way ANOVA followed by Tukey's test (GraphPad Prism 8).

The gastric protective effect of a set of synthesized compounds was evaluated through an indomethacin induced gastric ulcer model in rats. As negative control, animals of saline group were only given normal saline (5 mL/kg). The percentages of the ulcer inhibition, shows that the compounds tested had significant anti‐ulcer activity. The normal drug omeprazole showed an inhibition of 85.30 ±4 and therefore acted as a positive control. The most efficacy was observed in compounds 5i, 5e, and 5a, which showed an outstanding inhibition of 83.41 ± 3.5, 72.19 ± 3.4, and 71.28 ± 4.4 reduction of ulcer formation, significantly (*p* < 0.001). Compounds 5g and 5b gave moderate results with the percentage of inhibition being 51.48 ± 3.62 and 43.60 ± 3.3, respectively (Figure 16).

### 3.13 Determination of Myeloperoxidase (MPO) in the Gastric Mucosal Tissue

**FIGURE 17:**
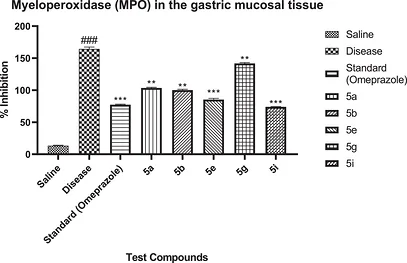
Determination of Myeloperoxidase (MPO) in the gastric mucosal tissue of rats. Findings were stated as mean ± SEM (*n* = 6/group). *p < 0.05, **p < 0.01, ***p < 0.001 versus omeprazole; #*p* < 0.05 versus ulcer control. Analysis by one‐way ANOVA followed by Tukey's test (GraphPad Prism 8).

To assess the anti‐inflammatory activity of the newly formed compounds, MPO activity was measured in gastric mucosal tissue following indomethacin‐induced ulceration. In the study described, the procedure was modified using the study [39]. Five experimental derivatives that is, 5a, 5b, 5e, 5g, and 5i were given through oral dose (10 mg kg) and compared with 30 mg kg dose of indomethacin. After the induction of gastric ulcers, tissue samples were taken, and MPO activity was measured using a spectrophotometric test and was estimated as units per gram of tissues (U/g tissue). In the absence of indomethacin, statistically notable rise in MPO activity of all derivatives compared to the control of indomethacin monotherapy, equating to a lower abundance of neutrophils and lower inflammation in the gastric wall occurred. Among the derivatives, 5i showed the greatest degree of inhibitory influence on MPO and, therefore, demonstrated the strongest indication of anti‐inflammatory property. This was followed by compounds 5e, 5b, 5a, and 5g in decreasing order of activity (Figure 17). These outcomes emphasize the therapeutic properties of selected derivatives in mitigating inflammation associated with gastric mucosal injury.

### 3.14 Determination of Malondialdehyde (MDA) Levels in Gastric Mucosal Tissue

Selected compounds 5a, 5b, 5e, 5g, and 5i were applied at a dose of 10 mg/kg and the omeprazole (30 mg/kg) served as a reference standard. After treatment, tissues of the stomach were homogenized and then analyzed using thiobarbituric acid reactive substances (TBARS) assay. Spectrophotometrically was used to measure MDA concentrations via recording the absorbance of the supernatant at 532 nm, and findings were stated as µmol MDA/g tissue (µmol/g tissue).

**FIGURE 18:**
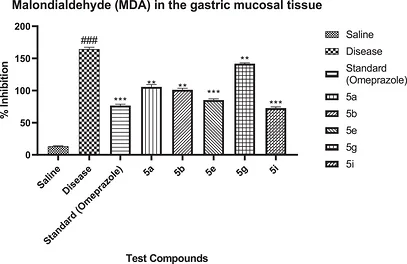
Determination of Malondialdehyde (MDA) in gastric mucosal tissue. Data are mean ± SEM (*n* = 6/group). *p < 0.05, **p < 0.01, ***p < 0.001 versus omeprazole, #*p* < 0.05 versus ulcer control. Analysis by one‐way ANOVA followed by Tukey's test (GraphPad Prism 8).

Compared to the indomethacin‐only control group, all selected benzimidazole‐pyrazole derivatives significantly reduced MDA levels, indicating their antioxidant potential in preventing lipid peroxidation. Among the tested compounds, 5i and 5e showed the most pronounced decrease in MDA levels, suggesting potent protective activity. Compounds 5b, 5a, and 5g also demonstrated moderate effectiveness. As expected, the standard drug omeprazole showed a notable decrease in melondialdehyde levels compared to the control group (Figure 18).

### 3.15 Biochemical Analysis of COX‐2 and TNF‐α in Rat Gastric Mucosa

**FIGURE 19:**
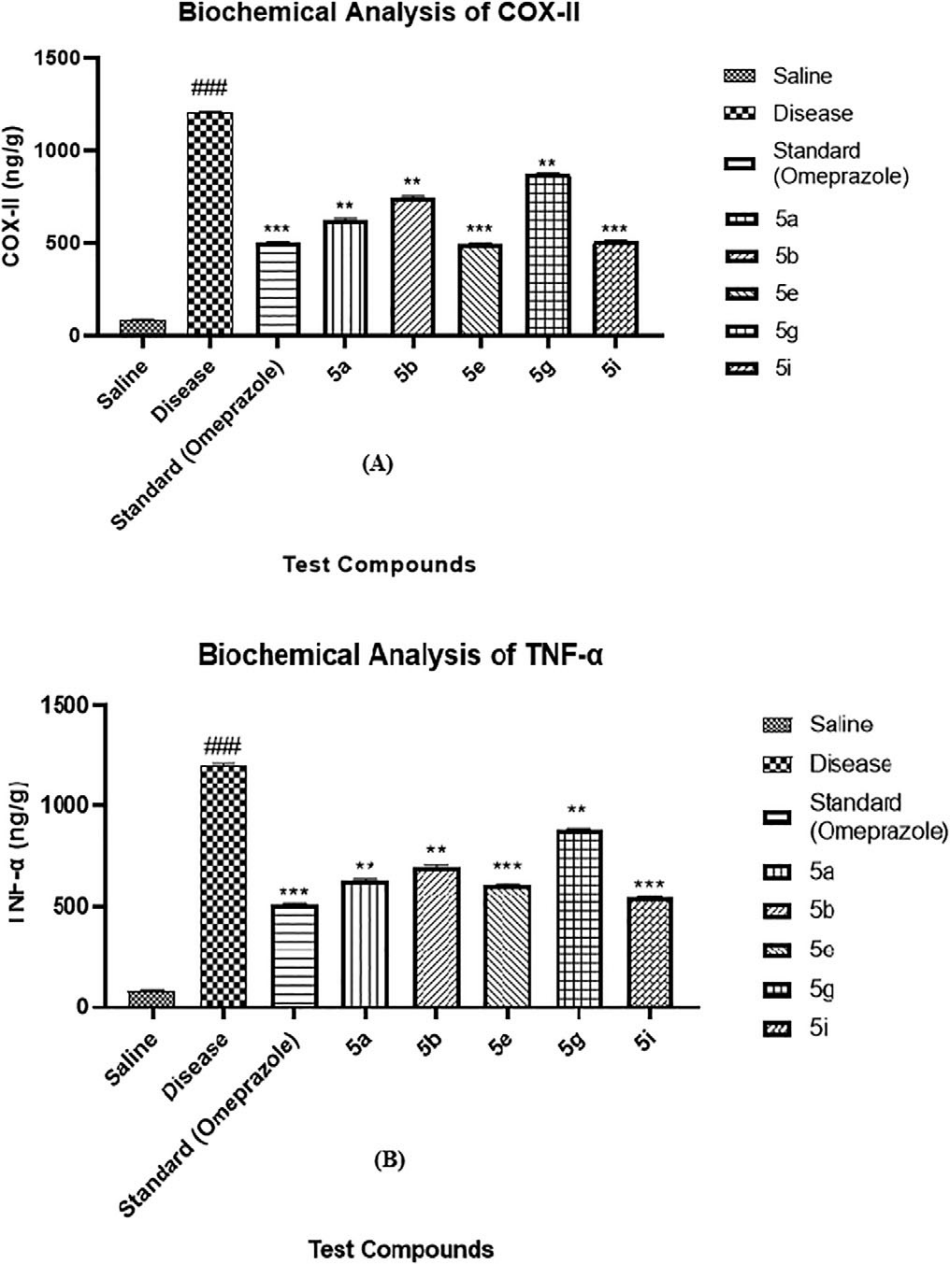
Effect of benzimidazole‐pyrazole hybrids and celecoxib on (A) COX‐2 and (B) TNF‐α in indomethacin‐induced gastric inflammation in rats. Data are mean ± SEM (*n* = 6/group). *p < 0.05, **p < 0.01, ***p < 0.001 versus negative control.

All tested compounds reduced TNF‐α levels, with compounds 5i and 5e exhibiting the strongest suppression (***p < 0.001) (Figure 19B). Regarding COX‐2 expression, compounds 5e and 5i significantly decreased their levels in gastric tissue (**p < 0.001) compared to the negative control group (Figure 19A). These results indicate that the synthesized benzimidazole‐pyrazole derivatives possess notable activity against inflammation by downregulating critical pro‐inflammatory cytokines TNF‐α and COX‐2 following indomethacin‐induced gastric injury.

### 3.16 Acute Oral Toxicity Studies of Benzimidazole‐Pyrazole Hybrids

In the present study, four sets of rats (*n* = 3) were formed. No treatment was performed on the control group (A) as all the others were under the same conditions. Group B, C, and D were treated with compounds 5a, 5e, and 5i, respectively, to a total dose of 100 mg/kg bodyweight. Rats were given water and a well‐balanced diet. The 0.9% saline solution of 1 mL/100gm body weight was administered per orally to the control group (Table 3). The experiment followed behavior patterns.

**TABLE 3:** Illustrates the observed behavioral patterns and toxicity‐related symptoms.

Observation	Group A(Control)	Group B (5a)	Group C (5e)	Group D (5i)
**Signs of illness**	−	−	−	−
**Ocular toxicity** **Eye Irritation**	−	−	−	−
**Lacrimation**	−	−	−	−
**Salivation**	−	−	−	−
**Convulsions**	−	−	−	−
**Touch response**	+	+	+	+
**Corneal reflex**	+	+	+	+
**Righting reflex**	+	+	+	+
**Gripping strength**	+	+	+	+
**Alertness**	+	+	+	+
**Mortality**	—	—	—	—

## 4 Discussion

Benzimidazole derivatives have been reported as potent H^+^/K^+^‐ATPase inhibitors with PPI‐like profiles, state‐of‐the‐art structural and biophysical studies on H^+^/K^+^‐ATPase provide a modern framework that rationalizes the favorable binding poses and dominant hydrophobic terms [40]. COX‐2 selectivity and anti‐inflammatory efficacy of pyrazole‐containing scaffolds, consistent with COX‐2 docking, anti‐inflammatory and contemporary pharmacology and gastroenterology studies reaffirm the centrality of COX‐2 signaling in mucosal protection and ulcer healing, including modulation of inflammasome pathways relevant to ethanol and NSAID injury mechanistic threads that resonate with TNF‐α, MPO, and MDA findings [41].

The present study addresses a persistent clinical need for novel anti‐ulcer agents with multi‐target efficacy and minimal side effects. Although the incidence and prevalence of PUD have increased globally, current therapeutic strategies remain limited by adverse effects, drug resistance, and poor long‐term outcomes, especially in NSAID and stress induced ulcers. Here, we report the successful production and assessment of benzimidazole‐pyrazole hybrids as dual‐action molecules targeting key inflammatory and proton pump pathways. Compounds 5i and 5e demonstrated superior docking affinities for both H^+^/K^+^ ATPase and COX‐2 enzymes. In order to investigate the possible molecular interactions of the synthesized benzimidazole‐pyrazole hybrids 5a–5j, molecular docking simulations were performed against H+/K+ ATPase (PDB ID: 5YLU) and Cyclooxygenase‐2 (COX‐2; PDB ID: 3LN1). The results from docking studies provided a structural hypothesis in the active site cavity of both proteins with favorable binding free energies (ΔG) when compared to the standard drugs omeprazole and celecoxib. The ΔG value for compound 5e was found to be −9.3 kcal/mol against COX‐2, the highest amongst the series, and much closer to the reference drug celecoxib having a ΔG value of −9.4 kcal/mol. Similarly, compound 5i exhibited the highest binding affinity against H^+^/K^+^ ATPase with a ΔG of −9.4 kcal/mol, much higher than the standard drug omeprazole with a ΔG of −8.2 kcal/mol. Analysis of two‐dimensional interaction diagrams showed the participation of hydrogen atoms of the amino group of the imidazole ring in hydrogen bonding with important active site residues. Apart from that, the aromatic rings of the hybrid molecules interacted through non‐covalent interactions like π‐sigma, π‐alkyl, and π‐donor hydrogen bonding with important amino acids inside the binding pocket of both the enzymes [42, 43]. In silico findings were further cross‐validated through MD simulations, showing that the conformational integrity of ligand‐receptor bonds remained preserved under physiological conditions regarding RMSD, hydrogen bonding, and compactness of protein‐ligand interface.

From a molecular perspective, the docking and MD simulations revealed that these compounds form stable complexes in the active sites of H^+^/K^+^‐ATPase and COX‐2, interacting with canonical residues through H‐bonding (imidazole‐NH donors) and π‐mediated contacts which stabilize the complexes. Indeed, these time‐dependent stability metrics, RMSD/RMSF, compactness (Rg), SASA, hydrogen‐bond occupancies, and MM/GBSA energies converged on a picture of well‐behaved, equilibrated complexes dominated by van der Waals contributions, as expected from hydrophobic cavity engagement in both targets [44]. Such findings are fully aligned with state‐of‐the‐art structural and computational insights into ligand recognition by H^+^/K^+^‐ATPase, including recent structural studies about ion‐binding states, and with ongoing medicinal chemistry studies on benzimidazole scaffolds and pyrazole‐based selective COX‐2 inhibitors.

It is crucial to understand that the computer studies should be regarded as hypothetical mechanisms, not as proof of the target inhibition. The H^+^/K^+^‐ATPase inhibition was tested in the laboratory, while the binding of the drug to the COX‐2 was evaluated using the molecular docking and MD simulations and decrease in the amount of COX‐2 in the gastric mucosa.

Pharmacologically, these compounds exerted potent gastroprotective effects in ethanol‐ and indomethacin‐induced ulcer models. The efficacy, often comparable to the standard drug omeprazole, was shown across several biochemical and histopathological parameters. Compound 5i was found to consistently show the most pronounced inhibition of ulcers and biochemical normalization, including marked suppression of pro‐inflammatory mediators (TNF‐α and COX‐2) and oxidative stress indicators (MDA and MPO). This indeed suggests a dual mechanism involving the inhibition of acid secretion and suppression of inflammation.

The inhibition of gastric H^+^/K^+^ ATPase activity by 5i and 5e supports further investigation of a PPI‐like mechanism and possibly provides enhanced acid suppression compared to the current PPIs, which have the disadvantages of slow onset and the development of tolerance. In addition, their ability to increase levels of endogenous PGE_2_ suggests a role in mucosal protection, often compromised by conventional NSAID therapy. Safety profiles from acute toxicity studies conducted on the compounds were good even at high doses. This further strengthens their candidature for possible clinical translation. The most active hybrids, 5i and 5e, functionally elicited reductions in ulcer burden against ethanol‐ and indomethacin‐induced models, with efficacy matching omeprazole and normalization of the TNF‐α, COX‐2, MPO, and MDA markers central to neutrophil‐driven inflammation and lipid peroxidation in the gastric mucosa. Inhibition of gastric H^+^/K^+^‐ATPase activity corroborates a PPI‐like mechanism, while the suppression of COX‐2/TNF‐α and improvement of oxidative stress indices support parallel anti‐inflammatory and antioxidant actions. Increased PGE2 further supports the restoration of mucosal defense, in line with the acknowledged cytoprotective role of PGE2 in maintaining blood flow, the output of mucus/bicarbonate, and epithelial restitution during ulcer healing.

The structure‐activity relationships (SARs) indicated that the nature and the position of the substituted groups on the benzimidazole‐pyrazole system influenced the biological activity. Compounds 5e and 5i proved to be pharmacologically important in this family of compounds, their profiles were quite different from each other in terms of target association; that is, 5e possessed the best COX‐2 binding properties, whereas 5i had the best H^+^/K^+^‐ATPase binding properties and gastro‐protection. It seems likely that the improved biological activity of these analogues is due to their ability to make favorable hydrophobic interaction and hydrogen bonding with the active site residues of H^+^/K^+^‐ATPase and COX‐2 enzymes as shown by docking and MD simulation studies. On the other hand, analogues with poor biological activity have been found to possess weak binding affinity and poor interactions with the key catalytic residues. The observed relationship between computed predictions and experimental results is an indication that the design of these analogues has merit, and the electronic and steric effects introduced into the benzimidazole‐pyrazole system have significant impact on their anti‐ulcer activities.

From the biological and computational viewpoints, the anti‐ulcer activity of benzimidazole‐pyrazole hybrids was shown to be significantly affected by electronic, steric, hydrophobicity and hydrogen‐bonding properties of the substituents on the aromatic system. Compounds 5e and 5i appeared to be highly informative SAR members despite some differences in their activity pattern. Compound 5e, having only one 4′‐methoxy (─OCH_3_) substituent, demonstrated the highest predicted affinity to COX‐2 with docking score of −9.3 kcal/mol, approaching the score of celecoxib (−9.4 kcal/mol). The predicted binding mode included Tyr324, Leu321/328, Trp356, Ala496, Val318/492, and Ser322 and had much in common with the binding mode of celecoxib, which suggested that mono‐methoxy substitution was able to provide a proper compromise between electronic tuning, hydrophobic properties and steric demands for binding into the COX‐2 binding site.

On the other hand, 5i is a compound with three methoxy substituents, thus introducing more oxygen atoms for hydrogen‐bond acceptor roles and considerable tuning of electronic, polar and spatial properties of the aromatic end. The increase in the number of methoxy groups seems to be associated with the ability of the ligand to recognize the binding site of H^+^/K^+^‐ATPase rather than linear rise in the activity against both targets. Compound 5i demonstrated the best predicted binding to H^+^/K^+^‐ATPase (−9.4 kcal/mol) in comparison to omeprazole (−8.2 kcal/mol), and the similar set of interacting residues including Glu64, Glu249, Ile16, and Ile306 as in the binding environment of the reference ligand. The extra methoxy groups can contribute to ligand‐protein recognition by increasing the number of hydrogen bond acceptors and making favorable polar interactions, whereas adding hydrophobic/aromatic area for van der Waals and π‐interactions at the same time. From medicinal chemistry point of view, it means that the role of the substituents is multidimensional: their electronic, hydrophobic and steric effects, along with hydrogen‐bonding properties determine the ability of the ligand to adopt a productive binding orientation into the certain binding site.

No signs of mortality or any other behavioral or clinical signs attributable to toxicity were observed within the 14‐day observation period after administration of 100 mg/kg. These data indicate some initial indications for tolerance to the given dosage of the compound; however, it does not indicate its overall toxicity profile. Subacute/chronic toxicity studies along with organ‐specific toxicity tests are necessary.

These results are in agreement with previous studies of benzimidazole‐based proton pump inhibitors and pyrazole‐containing COX inhibitors, but the combination of both motifs in one scaffold seems to confer a synergistic advantage. The demonstrated antioxidant activity may further reduce ethanol‐induced oxidative damage, often left unaddressed by conventional therapy. This makes the hybrids multi‐target agents that may effectively address the multifactorial etiology of peptic ulcer disease better than single‐target drugs. Despite these promising observations, several limitations need to be acknowledged. First, the major part of this study has focused on acute ulcer models, which might not be a full reflection of the chronic‐relapsing nature of peptic ulcer disease in humans. Second, though the docking and MD simulations provided a mechanistic view, further enzyme inhibition assays and biochemical studies have to be conducted for the establishment of dual‐target activity at the molecular level.

The results obtained from the experiments confirm the hypothesized multi‐target gastroprotection of the designed benzimidazole–pyrazole hybrids. The antigastric ulcer properties can be explained through the interaction of various activities such as inhibition of the gastric proton pump, inhibition of the inflammatory mediators, and oxidative stress mitigation. The strong inhibitory activity of compounds 5e and 5i on the H^+^/K^+^‐ATPase could be responsible for the decrease in the acidity of the stomach, while the decline in the levels of COX‐2 and TNF‐α would mean that the inflammatory mediators associated with gastric mucosa damage have been modulated. Also, the decrease in the levels of MPO and MDA indicates that there is a reduction in the inflammation and lipid peroxidation mediated by neutrophils.

## 5 Conclusion

In the present study, an integrated approach was made for designing, synthesizing, and evaluating a new series of benzimidazole‐pyrazole hybrids for anti‐ulcer activity on the basis of in silico, in vitro, and in vivo studies. Among the synthesized derivatives, compounds 5e and 5i exhibited favorable pharmacology results and require further study via pharmacokinetics, selectivity, and long‐term toxicity research before being considered as lead compounds due to their potent H^+^/K^+^ ATPase and COX‐2 enzyme inhibition with strong binding affinities, which were validated through molecular docking and dynamic simulations. These hybrids have shown remarkable gastroprotective effects against ethanol‐ and indomethacin‐induced ulcer models as evidenced by a significant reduction in ulcer indices and restoration of biochemical markers of oxidative stress and inflammation. The dual‐action mechanism‐acid suppression and anti‐inflammatory activity‐together with favorable safety profiles, positions these compounds as leads for the further development of safer and more effective anti‐ulcer agents. Further studies comprising chronic toxicity evaluation, pharmacokinetic profiling, and clinical investigations are warranted for fully realizing their therapeutic potential.

## Author Contributions

H.A.A.K. conceptualization, study design, methodology and validation of in silico studies. A.M. pharmacological activities. H.N. study design and methodology development, and supervision. M.S.O. contribution to methodology and validation. S.A. computational analyses and result interpretation. A.U.K. study design and methodology development, and supervision. M.A.F. pharmacological activities. H.M. manuscript designing, write‐up supervision. S.M.M. manuscript designing, computational analyses, result interpretation. All authors reviewed the manuscript.

## Funding

This research was supported by the Research Funding Program, King Saud University, Riyadh, Saudi Arabia under Grant No (ORF‐2026‐376).

## Conflicts of Interest

The authors declare no conflicts of interest.

## Supporting information



Supporting File 01: cbdv71698‐sup‐0001‐SuppMat.docx

## Data Availability

All relevant data supporting the findings of this study will be made freely available in the manuscript, supplementary file at the time of publication, without restriction. The repository will provide a permanent DOI for citation and long‐term access. The dataset will include all raw and processed data, NMR, HRMS, IR data to reproduce the results reported in this manuscript.
