# Supplementary material for: Computational, In Vitro, and In Vivo Evaluation of Benzimidazole–Pyrazole Hybrid Scaffolds as Multi‐Target Anti‐Ulcer Agents
Source: Chem Biodivers. 2026 Sep 15;23(9):e71698. doi: 10.1002/cbdv.71698 (PMC13575949; doi:10.1002/cbdv.71698)
Supplement: Supplementary file 1 [file CBDV-23-e71698-s001.docx]

**Figure S1:** Structures of the Synthesized Compounds (5a-5j)

**Scheme S1:** Synthesis of different chalcones C1-C10

**Scheme S2:** Synthesis of benzimidazole-pyrazole hybrids (5a-5j)

**Figure S2:** Structures of chalcones (C1-C10)

**Figure S3**: Structure of Benzimidazole-pyrazole hybrids

**Table S1.** FTIR data of different synthesized compounds

| Compounds | C=O | C=N | N-H | C=C | C-N | C-S | OH |
| --- | --- | --- | --- | --- | --- | --- | --- |
| 5a | 1655 | 1632 | 1585 | 1551 | 1137 | 1015 | - |
| 5b | 1663 | 1627 | 1590 | 1563 | 1115 | 1040 | - |
| 5c | 1671 | 1643 | 1567 | 1548 | 1151 | 1035 | 3387 |
| 5d | 1650 | 1636 | 1580 | 1562 | 1144 | 1021 | - |
| 5e | 1673 | 1623 | 1557 | 1549 | 1129 | 1005 | - |
| 5f | 1651 | 1630 | 1587 | 1561 | 1145 | 1080 | - |
| 5g | 1684 | 1653 | 1597 | 1569 | 1140 | 1032 | - |
| 5h | 1654 | 1634 | 1582 | 1550 | 1135 | 1024 | - |
| 5i | 1669 | 1648 | 1573 | 1565 | 1131 | 1090 | - |
| 5j | 1666 | 1628 | 1579 | 1547 | 1142 | 1055 | 3357 |

**Figure S4:** Fourier Transform Infrared Spectrum of compound C7

**Figure S5:** Fourier Transform Infrared Spectrum of compound

Intermediate 2

**Figure S6:** Fourier Transform Infrared Spectrum of compound C5

**Figure S7:** Fourier Transform Infrared Spectrum of compound C1

**Figure S8:** Fourier Transform Infrared Spectrum of compound 5a

**Figure S9:** Fourier Transform Infrared Spectrum of compound 5b

**Figure S10:** Fourier Transform Infrared Spectrum of compound 5c

**Figure S11:** Fourier Transform Infrared Spectrum of compound 5d

**Figure S12:** Fourier Transform Infrared Spectrum of compound 5e

**Figure S13:** Fourier Transform Infrared Spectrum of compound 5i

**Figure S14:** ^1^HNMR Spectrum of compound 5a

**Figure S15:** ^1^HNMR Spectrum of compound 5b

**Figure S16:** ^1^HNMR Spectrum of compound 5c

**Figure S17:** ^1^HNMR Spectrum of compound 5d

**Figure S18:** ^1^HNMR Spectrum of compound 5e

**Figure S19:** ^1^HNMR Spectrum of compound 5f

**Figure S20:** ^1^HNMR Spectrum of compound 5g

**Figure S21:** ^1^HNMR Spectrum of compound 5h

**Figure S22:** ^1^HNMR Spectrum of compound 5i

**Figure S23:** ^1^HNMR Spectrum of compound 5j

**Figure S24:** ^13^C NMR Spectrum of compound 5a

**Figure S25:** ^13^C NMR Spectrum of compound 5b

**Figure S26:** ^13^C NMR Spectrum of compound 5c

**Figure S27:** ^13^C NMR Spectrum of compound 5d

**Figure S28:** ^13^C NMR Spectrum of compound 5e

**Figure S29:** ^13^C NMR Spectrum of compound 5f

**Figure S30:** ^13^C NMR Spectrum of compound 5g

**Figure S31:** ^13^C NMR Spectrum of compound 5h

**Figure S32:** ^13^C NMR Spectrum of compound 5j

**Figure S33:** HRMS Spectrum of compound 5d

**Figure S34:** HRMS Spectrum of compound 5e

**Figure S35:** HRMS Spectrum of compound 5h

**Figure S36:** HRMS Spectrum of compound 5j
